# High-Resolution Genetic Map for Understanding the Effect of Genome-Wide Recombination Rate on Nucleotide Diversity in Watermelon

**DOI:** 10.1534/g3.114.012815

**Published:** 2014-09-15

**Authors:** Umesh K. Reddy, Padma Nimmakayala, Amnon Levi, Venkata Lakshmi Abburi, Thangasamy Saminathan, Yan. R. Tomason, Gopinath Vajja, Rishi Reddy, Lavanya Abburi, Todd C. Wehner, Yefim Ronin, Abraham Karol

**Affiliations:** * Gus R. Douglass Institute, Department of Biology, West Virginia State University, Institute, West Virginia 25112-1000; † U.S. Vegetable Laboratory, USDA-ARS, 2875 Savannah Highway, Charleston, South Carolina 29414; ‡ Department of Horticultural Science, North Carolina State University, Raleigh, North Carolina 27695-7609; § Institute of Evolution, Haifa University, Haifa 31905, Israel

**Keywords:** high-density genetic map, genotyping by sequencing, genome-wide recombination rate, linkage disequilibrium, selective sweep, watermelon

## Abstract

We used genotyping by sequencing to identify a set of 10,480 single nucleotide polymorphism (SNP) markers for constructing a high-resolution genetic map of 1096 cM for watermelon. We assessed the genome-wide variation in recombination rate (GWRR) across the map and found an association between GWRR and genome-wide nucleotide diversity. Collinearity between the map and the genome-wide reference sequence for watermelon was studied to identify inconsistency and chromosome rearrangements. We assessed genome-wide nucleotide diversity, linkage disequilibrium (LD), and selective sweep for wild, semi-wild, and domesticated accessions of *Citrullus lanatus* var. *lanatus* to track signals of domestication. Principal component analysis combined with chromosome-wide phylogenetic study based on 1563 SNPs obtained after LD pruning with minor allele frequency of 0.05 resolved the differences between semi-wild and wild accessions as well as relationships among worldwide sweet watermelon. Population structure analysis revealed predominant ancestries for wild, semi-wild, and domesticated watermelons as well as admixture of various ancestries that were important for domestication. Sliding window analysis of Tajima’s *D* across various chromosomes was used to resolve selective sweep. LD decay was estimated for various chromosomes. We identified a strong selective sweep on chromosome 3 consisting of important genes that might have had a role in sweet watermelon domestication.

Watermelon belongs to the genus *Citrullus* Schrad. Ex Eckl. et Zeyh., which thrives in the Kalahari Desert (Namibia and Botswana) and is indigenous to southern Africa (Whitaker and Bemis 1976). The genus comprises four known diploid (n = 11) species (Dane and Liu 2007; Reddy *et al.* 2013). Among them is the annual *Citrullus lanatus* (Thunb.) Matsum et Nakai, which is indigenous to arid sandy regions of southern Africa (Meuse 1962; Robinson and Decker-Walters 1999). *C. lanatus* var. *lanatus* Schrad. Ex Eckl. et Zeyh and *C. lanatus* var. *citroides* (L.H. Bailey) are two botanical varieties (Levi *et al.* 2013). *C. lanatus* var. *lanatus* includes the wild and semi-wild *mucosospermus* (egusi types) and sweet *vulgaris* forms. The wild *mucosospermus* forms and the Tsamma types (*citroides*) look similar, except that in var. *lanatus*, the stomata have one pair of subsidiary cells as compared with three pairs in the Tsamma melon (Botha 1982). However, the types are quite diverse at the molecular and cytological levels (Nimmakayala *et al.* 2010; Reddy *et al.* 2013).

The Plant Genetic Resources Conservation Unit (PGRCU; Griffin, GA), US Department of Agriculture–Agricultural Research Services (USDA-ARS), maintains more than 1650 US plant introductions of *Citrullus lanatus var. lanatus* (Levi *et al.* 2013). Nimmakayala *et al.* (2014) performed the most recent diversity analysis using 134 single nucleotide polymorphisms (SNPs) from 130 cultivars from Africa, Asia, Europe, and the Americas and concluded seven different clusters, with no clear distinction of accessions by collection site or geographical identity. These findings agreed with those of previous studies (Levi *et al.* 2001, 2013; Nimmakayala *et al.* 2011; Romão 2000; Zhang *et al.* 2012) concluding a molecular diversity of 2–4% for cultivated watermelon.

Although we previously sampled 130 accessions of watermelon, we did not include wild and semi-wild forms of var. *lanatus* and therefore could not address major population–genetic inferences important to association genetics study. In the current study, we analyzed genome-wide diversity using a larger SNP dataset involving a robust collection of representative cultivated, wild, and semi-wild watermelon accessions from across the world. Unlike most studies focused on building maps with mapping populations developed from *lanatus* and *citroides* (Nimmakayala *et al.* 2014; Ren *et al.* 2012; Sandlin *et al.* 2012), we used a mapping population derived from a cross between sweet and unsweet accessions belonging to *C. lanatus* var. *lanatus*.

Genotyping by sequencing (GBS) is a next-generation sequencing-based method that takes advantage of reduced representation to allow for high-throughput genotyping of large numbers of individuals with a large number of SNP markers (Glaubitz *et al.* 2014; Sonah *et al.* 2013). The relatively straightforward, robust, and cost-effective GBS protocol is being applied to numerous species (Elshire *et al.* 2011; Glaubitz *et al.* 2014). Previous studies of barley and wheat (Mascher *et al.* 2013; Poland *et al.* 2012; Sim *et al.* 2012) demonstrated the use of SNPs generated by GBS technology to build high-density genetic maps. Poland *et al.* (2012) stressed the importance of high-density maps in defining collinearity with existing physical maps and for providing valuable tools for anchoring and ordering the whole-genome sequence. Inconsistencies in the currently available watermelon whole-genome sequence or future sequencing efforts targeting the whole genome of watermelon require a genome-wide high-density map for assembly. Such a map would be useful for revealing minor alterations that occur because of inversions and translocations in the genomes of diverse collections of watermelon. Also, a collinearity study of genetic and physical maps would help resolve inconsistencies resulting from the resequencing efforts in various watermelon accessions.

Poland *et al.* (2012) indicated the importance of high-density maps contributing to fundamental knowledge about genome structure. Such maps are also needed for genomic research into haplotypic imputation of missing data and integration with whole-genome shotgun sequencing contigs and BAC-end sequences to anchor and order the reference genomes. Combining whole-genome resequencing and genome-wide association study (GWAS) would help identify markers for quantitative trait loci (QTL); however, recombination mapping is still needed for validating previously identified markers, identifying new markers, and map-based cloning approaches.

Association mapping with diverse genotypes in plants is a new and powerful tool with promising results for identifying functional variation in both known and unknown genes associated with important agronomic and economic traits (Yan *et al.* 2009). Linkage disequilibrium (LD) is a key factor in determining the number of markers needed for GWAS and genomic selection. LD breakdown is affected by many genetic and nongenetic factors, including recombination, drift, selection, mating patterns, and admixture (Flint-Garcia *et al.* 2003; Yan *et al.* 2009; Yu and Buckler 2006). In this study, we aimed to characterize genome-wide LD in the watermelon genome and understand how LD is influenced by recombination rate and selective sweep.

In terms of genomic prediction or GWAS, understanding the landscape of recombination is of interest because the linkage phase between the marker and favorable QTL allele is crucial when predicting breeding values across diverse gene pools (Bauer *et al.* 2013; de Roos *et al.* 2009; Ren *et al.* 2012). In this study, we sought to associate the genome-wide variation in recombination rate (GWRR) and nucleotide diversity across the watermelon genome.

## Materials and Methods

We included 86 accessions of *C. lanatus* var. *lanatus* representing 22 wild, 13 semi-wild (egusi), and 51 sweet watermelons from a worldwide geographical area (Supporting Information, Table S1). To build a genetic map, 113 F_2_ progenies were obtained from a single F_1_ plant of a cross between accessions PI#482362 (egusi type), a white-flesh unsweet watermelon from Zimbabwe, and PI#270306 (sweet watermelon; plant ID, Mangara), a red-flesh watermelon from Zaire, kindly provided by Dr. Robert Jarret (PGRCU, USDA-ARS, Griffin, GA).

### SNP identification with GBS

Genomic DNA isolation involved use of the DNeasy plant mini kit (QIAGEN, Germany) and GBS followed the protocol of Elshire *et al.* (2011). Briefly, genome complexity was reduced by digesting total genomic DNA from individual samples with use of the *Ape*KI methylation-sensitive restriction enzyme. A suitable restriction enzyme for watermelon is *Ape*KI, a type II restriction endonuclease that recognizes a degenerate 5-bp sequence (GCWGC, where W is A or T), which creates a 5′ overhang (3 bp) and is partially methylation-sensitive (will not cut if the 3′ base of the recognition sequence on both strands is 5-methylcytosine). Digested products were then ligated to adapter pairs with enzyme-compatible overhangs; one adapter contained the barcode sequence and a binding-site Illumina sequencing primer (Illumina Inc., USA). Then, samples were pooled, purified, and amplified with primers compatible with the adapter sequences. Temperature cycling consisted of 72° for 5 min, 98° for 30 s, followed by 18 cycles of 98° for 30 s, 65° for 30 s, and 72° for 30 s, with a final *Taq* extension step at 72° for 5 min. These amplified sample pools constitute a sequencing “library.” Libraries were purified and 1 µL was loaded onto an Experion automated electrophoresis station (BioRad, Hercules, CA) for evaluation of fragment sizes. Libraries were considered suitable for sequencing if adapter dimers (∼128 bp in length) were minimal or absent and most of the other DNA fragments were between 170 and 350 bp. If adapter dimers were present in excess of 0.5% (based on the Experion output), libraries were constructed again by using a few DNA samples and decreasing adapter amounts. The PCR primers also added 3′ sequences complementary to the solid-phase olignucleotides that coat the Illumina sequencing flow-cell. After PCR, pooled products were purified; GBS “library” fragment size distributions were checked on a BioAnalyzer (Agilent Technologies, Inc., USA). Products were quantified and diluted for sequencing by use of Illumina HiSequation 2500. A bioinformatics pipeline, TASSEL-GBS, designed for efficient processing of raw GBS sequence data into an SNP genotype file (Glaubitz *et al.* 2014) was used. Barcoded sequence reads were processed and collapsed into a set of unique sequence tags, with one TagCounts file produced per input FASTQ. Chromosomal assignment and position on the physical map of candidate genes, GBS markers, were deduced by using the draft whole-genome sequence for watermelon (www.icugi.org).

### Genetic diversity and population structure analysis

To determine the appropriate population structure in the collection, we used different methodologies and software packages (Nimmakayala *et al.* 2014). For quantitative assessment of the number of groups in the panel, we used Bayesian clustering analysis with a model-based approach implemented in STRUCTURE v2.2 (Pritchard *et al.* 2000). This approach involves use of multi-locus genotypic data to assign individuals to k clusters or groups without prior knowledge of their population affinities. The program was run for k-values 1 to 9, with 100,000 burn-in iterations, followed by 500,000 Markov Chain Monte Carlo iterations for accurate parameter estimates with a high-performance cluster. To verify the consistency of the results, we performed three independent runs for each k. An admixture model with correlated allele frequencies was used. The optimal k value was determined by use of an *ad hoc* statistic, Δk (Evanno *et al.* 2005). The number k in each dataset was evaluated by Δk values estimated with the software Structure Harvester to visualize STRUCTURE output and implement the Evanno method. In a second approach, we performed principal component analysis (PCA) with the SNP and Variation Suite (SVS v7.7.6) (Golden Helix, Inc., Bozeman, MT, www.goldenhelix.com). In a third approach, we constructed a neighbor-joining (NJ) dendrogram based on Nei’s genetic distance matrix by using TASSEL v3.0 (http://www.maizegenetics.net) (Bradbury *et al.* 2007).

### Analysis of nucleotide diversity and selective sweep differentiation between domesticated and semi-wild watermelon

Observed nucleotide diversity (π), expected nucleotide diversity (θ), and Tajima’s *D* were estimated by using TASSEL v3.0 with a sliding-window approach representing π, θ, and Tajima’s *D* along all 11 watermelon chromosomes, similar to that described previously (Guo *et al.* 2013; Korneliussen *et al.* 2013). To identify potential selective sweep, we compared nucleotide diversity between populations of modern cultivars (*C. lanatus* var. *vulgaris*) and semi-wild accessions (*C. lanatus* var. *mucosospermus*).

### Construction of a large-scale genetic map with SNP markers

Linkage analysis and map construction of SNPs generated by GBS involved use of MultiPoint (http://www.multiqtl.com) based on reduction of the mapping problem to the traveler salesperson and solution heuristic algorithms based on Evolutionary Strategy optimization (Korol 2009; Mester *et al.* 2003, 2004). GBS resulted in a disproportion between the high number of scored markers for the mapping populations and population size. MultiPoint analysis allows for selecting the most informative markers for building a reliable skeletal map, whereas other markers are anchored to a skeletal framework map (Mester *et al.* 2003). For building a skeleton map, we selected error-free markers based on the presence of “twins” (*i.e.*, markers with zero distance) in the dataset. This approach derives from the expectation that because of genotyping errors, the probability of finding false recombinants between absolutely linked markers is higher than observing absolute linkage for closely (but not absolutely) linked markers. The major steps of the algorithm for building ultra-dense genetic maps implemented in MultiPoint include: a “delegate” marker selected from each twin group (including markers with zero distance); except for the twins of various groups, all remaining markers are moved to a heap; delegate markers are ordered to linkage groups (LGs); possible gaps in the LGs are filled by using markers from the heap that belong to twin groups of lower size or singleton markers; and map stability is tested by jack-knife resampling followed by removal of markers violating local map stability and/or monotony (*i.e.*, deviation from the expected increase of recombination rate between a marker and its subsequent neighbors along the map). The last step attaches the markers from the heap to the skeletal map. Each heap marker is attached to the skeletal map if its distance to the closest interval does not exceed the length of this interval. The genetic linkage map was graphically displayed by use of MapChart2.2 (Voorrips 2002).

### Recombination landscape

The recombination landscape was revealed by estimating the GWRR (in cM per Mbp) with the physical position of the watermelon genome sequence assembly. The number of recombination events per individual were estimated chromosome-wise by using Monte Carlo EM Cycle of Gibbs sampling available in the maximum likelihood mapping algorithm of JoinMap v4 (Van Ooijen 2006).

## Results

### SNP identification and molecular diversity and population structure

The sequencing of the *Ape*KI GBS libraries yielded 182 million reads per lane, before any processing. The TASSEL-GBS pipeline for identifying and calling SNPs allowed for simultaneous SNP discovery from various samples. A total of 67,897 SNPs were initially identified; 13,693 SNPs were selected by a cutoff of minor allele frequency (MAF) ≥0.01. In total, 10,370 of 13,693 SNPs genotyped had a call rate of >85%. Chromosomes 1 to 11 contained 1061, 1069, 1045, 570, 1043, 903, 776, 651, 950, 801, and 775 SNPs, respectively. A set of 726 SNPs could not be assigned to any chromosome. A total of 1563 SNPs survived filtering at MAF ≥0.05, with deviation from Hardy Weinberg equilibrium (at *P* > 0.01) and LD pruning (at 0.05) to remove identical SNPs, for a final 190, 172, 107, 81, 162, 160, 146, 118, 152, 139, and 137 SNPs on chromosomes 1 to 11, respectively.

**Figure 1:**
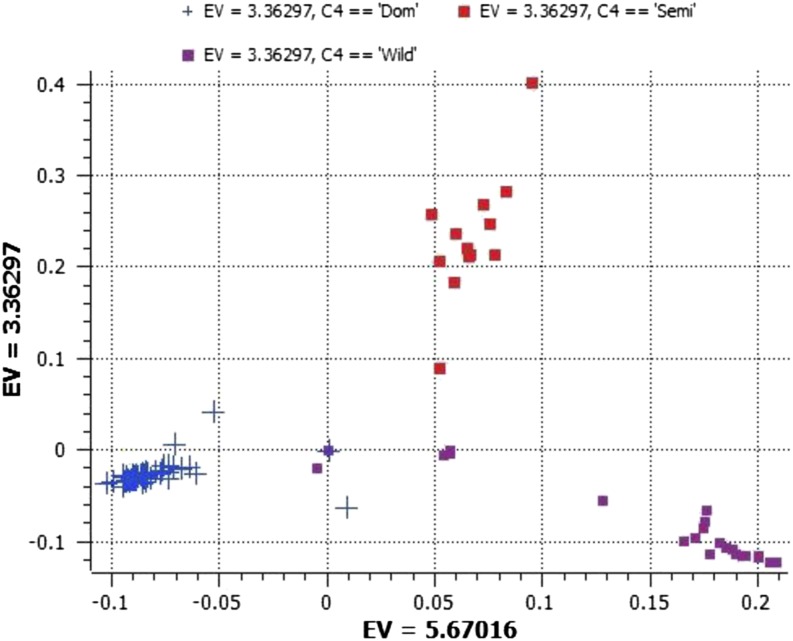
Principal component analysis (PCA) based on the first two components showing distribution of sweet, semi-wild, and wild watermelons by using 1563 single nucleotide polymorphisms (SNPs) generated by genotyping by sequencing (GBS). See Table S1 for a list of accessions and Table S2 for respective eigen values to locate individual accessions on the graph.

PCA of the 1563 SNPs revealed two dimensions, clustering according to cultivated, semi-wild, and wild accessions (Figure 1 and Table S2). We constructed 11 NJ trees with various chromosome-specific SNPs separately to resolve the differences among sweet, semi-wild, and wild watermelon and to understand the effect of various chromosome-specific SNPs on the clustering pattern. All chromosome-specific phylograms clearly separated wild, semi-wild, and sweet watermelon types into distinct clusters, so domestication of sweet watermelon is a genome-wide process. Chromosome-specific trees resolved sweet watermelons into a variable number of subclusters ranging from 2 to 10 (Figure S1A and Figure S1B). Despite no clear pattern of clustering based on geographic distribution, most of the US cultivars were grouped into a subcluster in all chromosome-specific trees.

Observed nucleotide diversity was mapped against the physical map to show the pattern of distribution. Nucleotide diversity varied within and across the chromosomes. Mean and SD of nucleotide diversity (observed and expected) for cultivated and semi-wild watermelon are provided in Table 1. The difference in nucleotides between semi-wild and cultivated watermelon was largest for the chromosome 3 as compared with the other chromosomes, so this chromosome harbors mutations and genes of importance for the process of domestication. Moreover, nucleotide diversity was the least in chromosome 3, which supports this chromosome harboring signals of domestication (Figure 5).

**Table 1:** Mean nucleotide diversity (observed π and expected θ) among semi-wild and cultivated watermelon across various chromosomes

Type	Chr. 1	Chr. 2	Chr. 3	Chr. 4	Chr. 5	Chr. 6	Chr. 7	Chr. 8	Chr. 9	Chr. 10	Chr. 11
Wild π	0.17 ± 0.03	0.17 ± 0.04	0.22 ± 0.07	0.18 ± 0.04	0.18 ± 0.03	0.16 ± 0.04	0.18 ± 0.04	0.19 ± 0.03	0.18 ± 0.04	0.16 ± 0.04	0.17 ± 0.03
Cult. π	0.15 ± 0.04	0.14 ± 0.05	0.11 ± 0.04	0.15 ± 0.04	0.16 ± 0.04	0.17 ± 0.05	0.17 ± 0.04	0.17 ± 0.03	0.17 ± 0.05	0.16 ± 0.04	0.15 ± 0.04
Wild θ	0.21 ± 0.02	0.22 ± 0.02	0.23 ± 0.02	0.22 ± 0.01	0.22 ± 0.01	0.20 ± 0.04	0.21 ± 0.02	0.22 ± 0.02	0.22 ± 0.02	0.20 ± 0.03	0.22 ± 0.01
Cult. θ	0.20 ± 0.03	0.19 ± 0.05	0.17 ± 0.05	0.20 ± 0.04	0.21 ± 0.01	0.21 ± 0.01	0.21 ± 0.01	0.21 ± 0.01	0.20 ± 0.02	0.20 ± 0.01	0.20 ± 0.02

Data are mean ± SD. Cult., cultivated; Wild, semi-wild.

**Figure 2:**
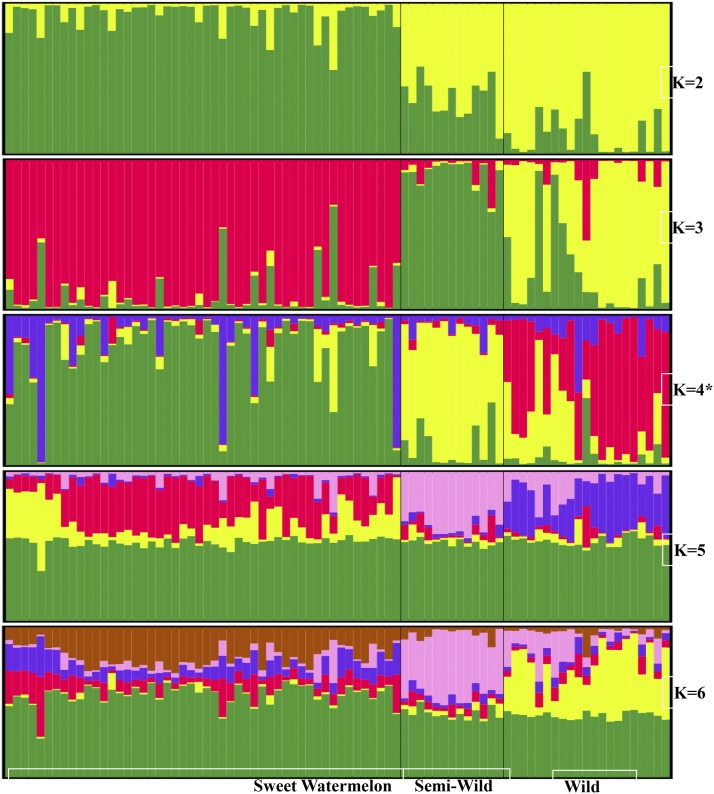
Population structure for k = 4. Clusters are separated by vertical lines with cluster colors indicating various ancestries. Red was predominant in wild, yellow was predominant in semi-wild, and green was predominant in cultivated watermelon. Cultivated watermelon represented most of the green and, to a lesser extent, purple, yellow, and red clusters.

We used a model-based approach to population structure analysis to analyze the entire panel of 86 sweet watermelon accessions (Figure 2). Mean LnP(K) and ΔK values are in Figure S2. K-4 was the most appropriate clustering for this population, with ΔK value 1100. We used the population structure to analyze ancestry rather than clustering. The ancestry distribution of K-4 (red, yellow, purple, and green) revealed its origin from wild-type watermelons. Red was predominant in wild, yellow was predominant in semi-wild, and green was predominant in cultivated watermelon. Cultivated watermelon represented most of the green and, to a lesser extent, purple, yellow, and red ancestry.

### High-density genetic map

**Figure 3:**
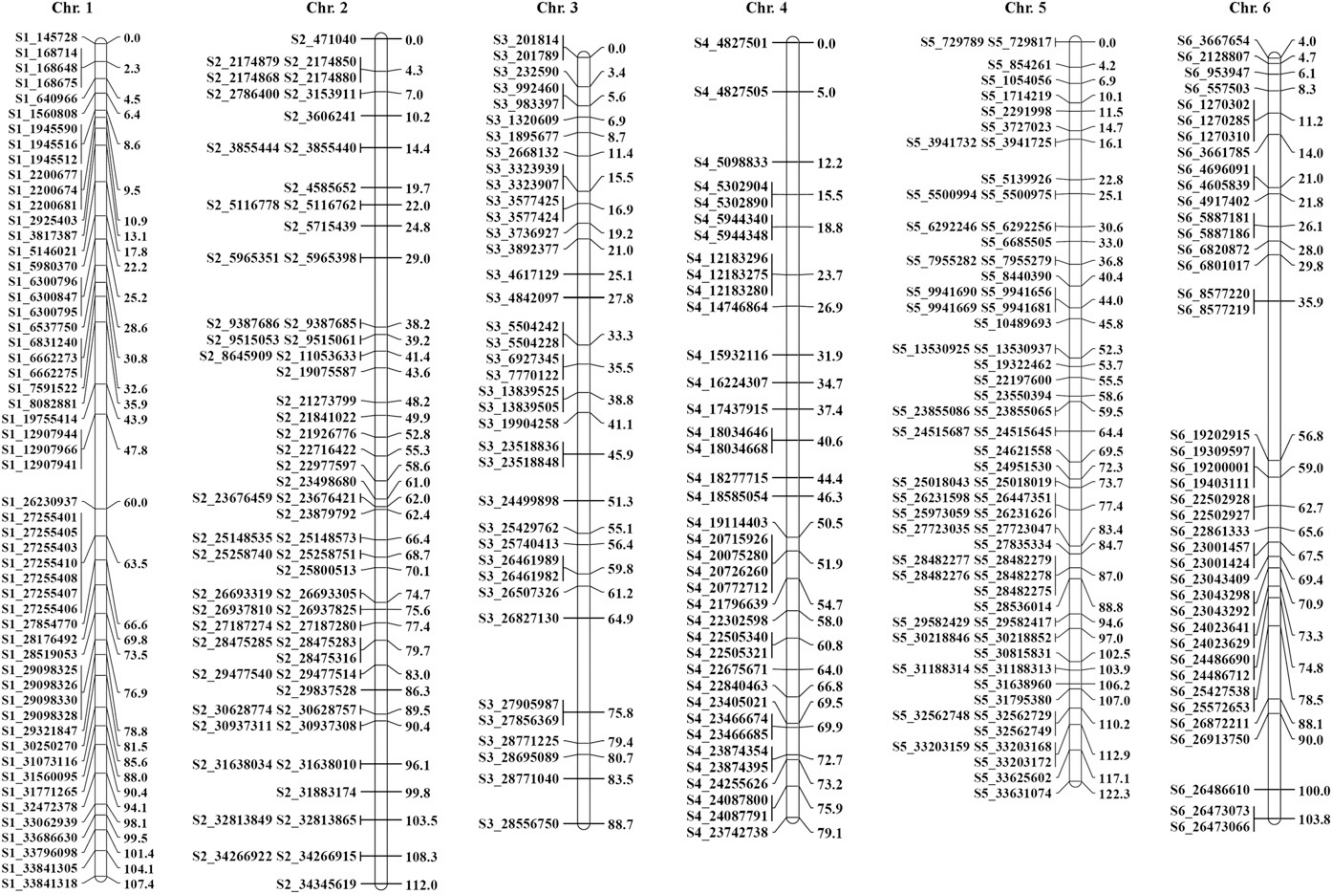

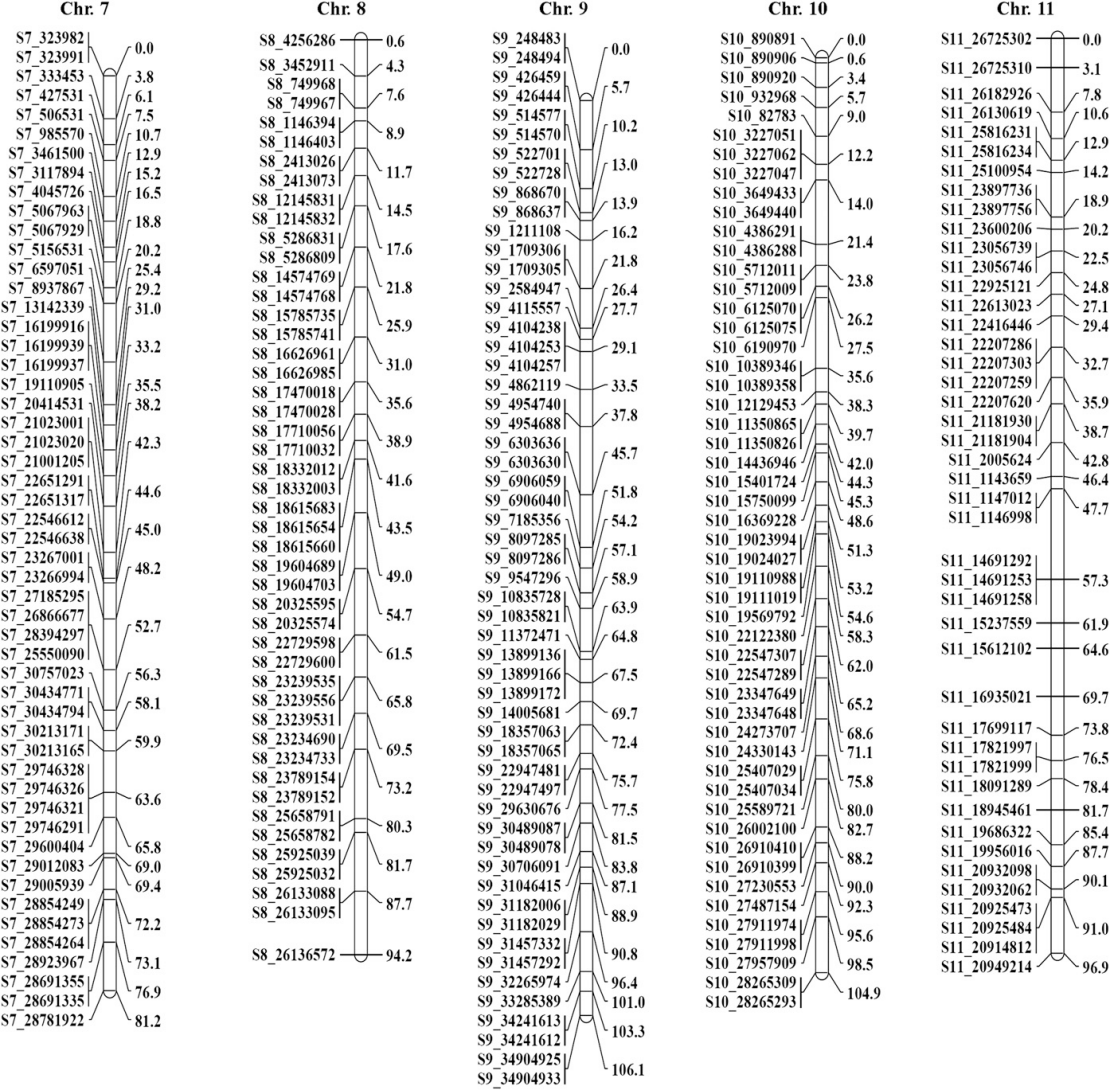
(A and B) Genetic map showing positions of skeletal markers on which a high-density genetic map is constructed.

We mapped 10,480 SNPs into a genetic linkage map using a mapping population that contained 113 progenies generated from a cross of egusi and sweet watermelon. Chromosome distribution of 547 skeletal markers is provided in Figure 3, A and B. To select skeletal markers, SNPs violating map stability on mapping were removed and linkage groups were reanalyzed several times until the map showed complete stability. Use of MultiPoint allowed for detection and removal of markers violating the order stability and monotonic growth of distances in the skeleton map. After cleaning, markers from the heap were checked as candidates for filling-in the gaps. The map showed a strong threshold of the absolute linked markers and showed very good correspondence between the map characteristics (the number of skeletal markers and length of the map). Chromosomes 1 to 11 contained 55, 61, 38, 38, 66, 40, 52, 47, 55, 51, and 44 skeletal markers, respectively, with genetic lengths (cM) 107.4, 112, 88.7, 79.1, 122.3, 103.8, 81.2, 94.2, 106.1, 104.9, and 96.9, respectively (Figure 3, A and B). In addition, the current map defined 3821 recombination events within the skeletal map. The skeletal map for chromosomes 1 to 11 contained 406, 339, 240, 219, 450, 257, 305, 391, 464, 373, and 388 recombination events, respectively. Each recombination bin or skeletal marker segregated with multiple add-on markers, for a high-density genetic map. Of note, chromosomes 3, 4, and 6 contained the least skeletal markers and fewer recombination events as compared with the other chromosomes, perhaps because of recombination suppression. In contrast, chromosomes 5, 2, and 9 possessed multiple recombination bins, so they contained hot spots of recombination. The entire length of the genetic map was 1096.53 cM. Keeping the framework markers as anchors, 9933 add-on SNPs were incorporated across the lengths of various chromosomes, for a high-density genetic map. Add-on SNPs were 1171, 819, 766, 558, 1332, 822, 874, 644, 1054, 1023, and 870 on chromosomes 1 to 11, respectively, for a high-density genetic map (add-on markers anchored to skeletal markers are in Supplementary Materials). Clearly, a large set of the remaining add-on markers could also be attached to the corresponding interval or marker on the skeleton map (Figure S3-1, Figure S3-2, Figure S3-3, Figure S3-4, Figure S3-5, Figure S3-6, Figure S3-7, Figure S3-8, Figure S3-9, Figure S3-10, and Figure S3-11). Total add-on or anchor markers are in Table S3, Table S4, Table S5, Table S6, Table S7, Table S8, Table S9, Table S10, Table S11, Table S12, and Table S13. Skeletal markers are framework markers with high confidence.

**Figure 4:**
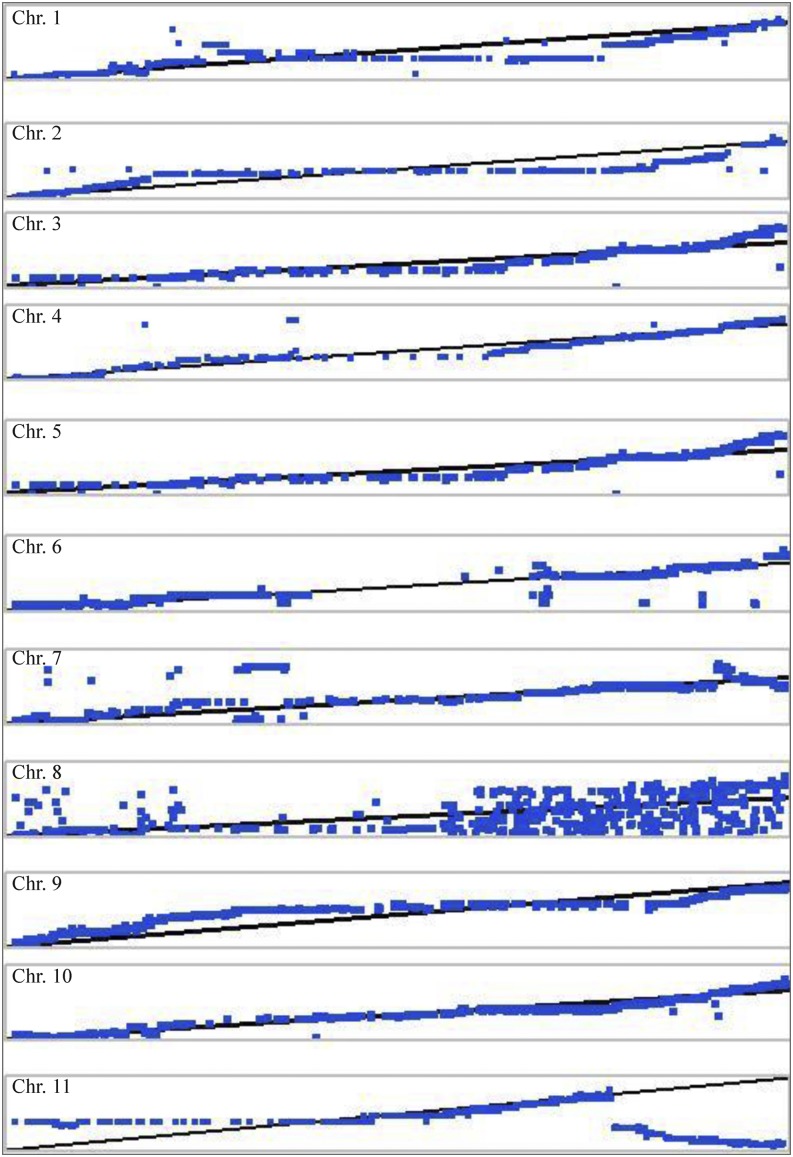
Collinearity between genetic and physical maps (markers that are distant from the “line of best fit” are not collinear).

We examined the collinearity of genetic and physical maps for various chromosomes (Figure 4). Markers on chromosomes 3, 5, 7, and 10 were highly co-linear in terms of physical location. Chromosomes 1, 2, 4, 8, and 9 moderately agreed with the watermelon reference sequence. Chromosome 11 showed the highest disagreement between the genetic and physical map on either side of the chromosome; it contained a large segment that was not collinear with the physical map. We observed 17 major genome rearrangements or disagreements across all chromosomes.

### GWRR

**Figure 5:**
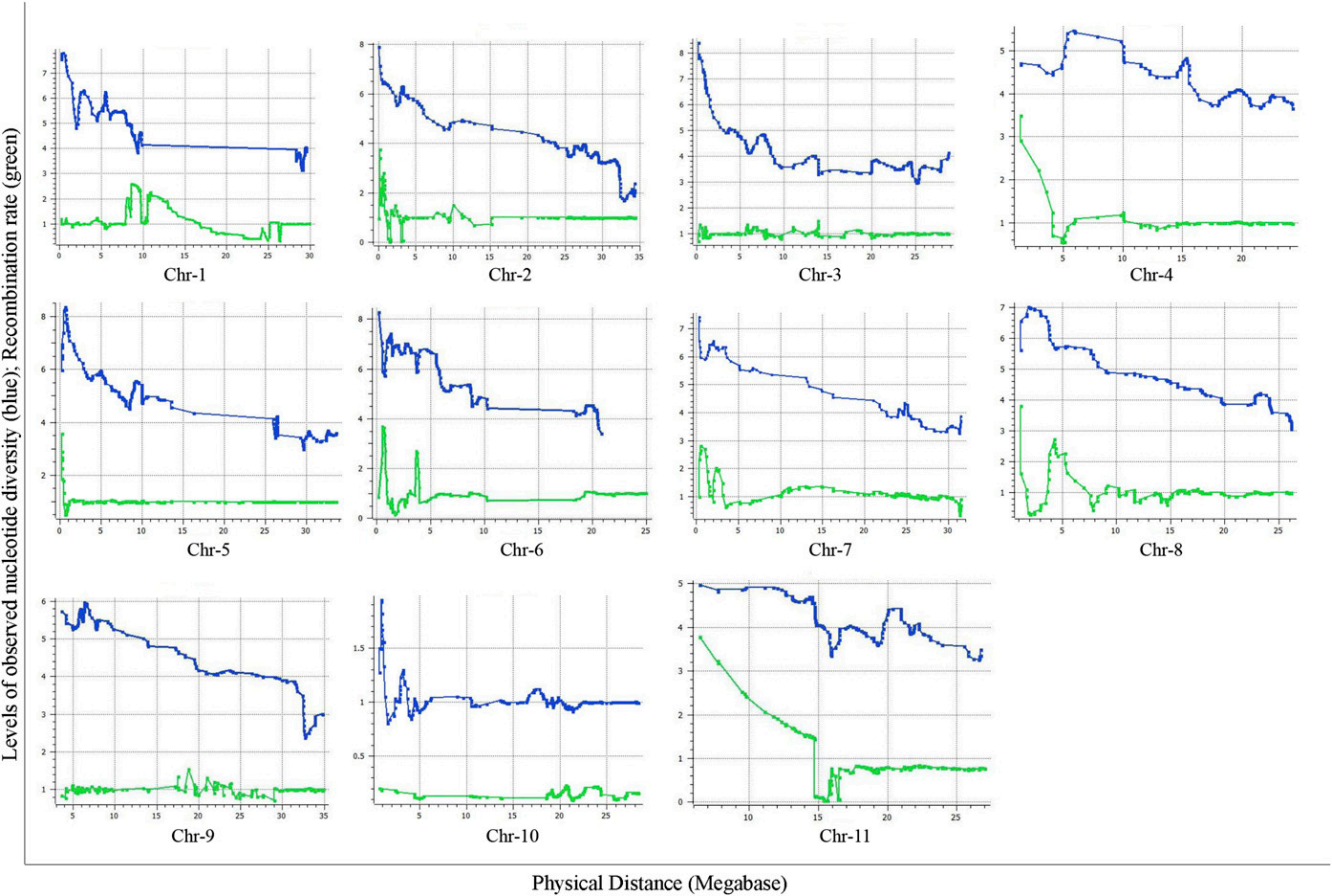
Distribution of genome-wide recombination rate (GWRR) and observed nucleotide diversity (π) along chromosomes in the watermelon genome. In each plot, the horizontal axis (in Mb) represents the physical distance (PD) along the reference chromosomes and the vertical axis (cM/Mb) represents the genetic-to-physical distance ratio (green) and log −2 transformed values of nucleotide diversity (π).

**Figure 6:**
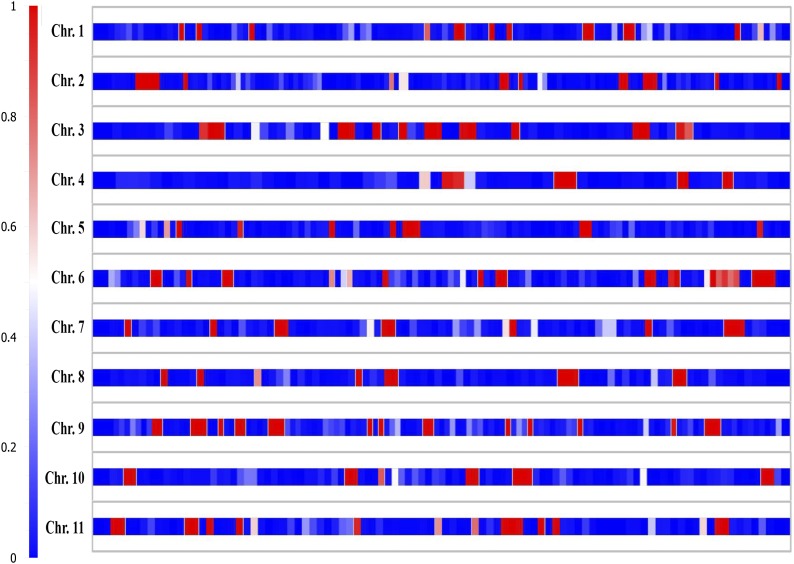
Linkage disequilibrium decay as measured by r^2^ averaged in distance intervals across the 11 watermelon chromosomes.

We estimated GWRR using the formula cM/Mb (Figure 5). We observed wide variation of GWRRs within and among the chromosomes. Mean GWRR for chromosomes 1 to 11 was estimated at 1.25, 1.09, 1.04, 1.25, 1.37, 1.34, 1.06, 1.18, 1.00, 1.15, and 1.49, respectively (Figure 6, A and B). GWRR ranges were 0.32–2.8, 0.03–3.8, 0.09–1.69, 0.28–3.6, 0.02–3.6, 0.21–3, 0.12–2.96, 0.12–3.85, 0.12–1.97, 0.03–3.45, and 0.04–3.80, respectively. Twelve hot spots of recombination containing GWRR in the range of 2 to 4 were distributed on chromosomes 1, 2, 4, 6, 7, 8, and 11 (Figure 5). Chromosomes 3, 5, 9, and 10 did not show GWRR >2, so this part of the genome may be less recombinant. However, a trend was noted whereby the hot spots of recombination (peak of GWRR) correspond to the increase in nucleotide diversity (π) on chromosomes 2, 4, 5, 6, 7, and 11 (Figure 5), so the recombination landscape was an important factor shaping the cultivar divergence on these chromosomes.

### Characterization of genome-wide LD

We conducted extensive LD analysis (Figure 6) showing that the extent and LD decay varied along chromosomes, with regions of high LD interspersed with regions of low LD. LD blocks are cold spots of recombination or spots of recombination suppression. We noted mean LD decay when testing SNPs with MAF of 0.05. Chromosomes 1 to 11 contained 11, 10, 10, 5, 9, 12, 7, 7, 13, 6, and 13 blocks with mean block size 1.43 Mb or 1.33 cM. The total lengths of LD blocks in cM were 14.77, 16.81, 18.20, 11.20, 9.0, 11.64, 9.54, 12.38, 19.63, 8.56, and 11.50 cM, respectively, with LD scaffold sizes (in Mb) 7.9, 6.18, 7.06, 4.09, 4.31, 5.84, 4.88, 3.50, 7.94, 6.62, and 7.78, respectively. Of note, the GWRR within blocks were 0.95, 0.86, 0.78, 0.93, 1.09, 0.99, 1.02, 0.94, 1.01, 1.00, and 0.96, respectively, as compared with the mean GWRR (1.2).

### Characterization of selective sweep and domestication signature

**Figure 7:**
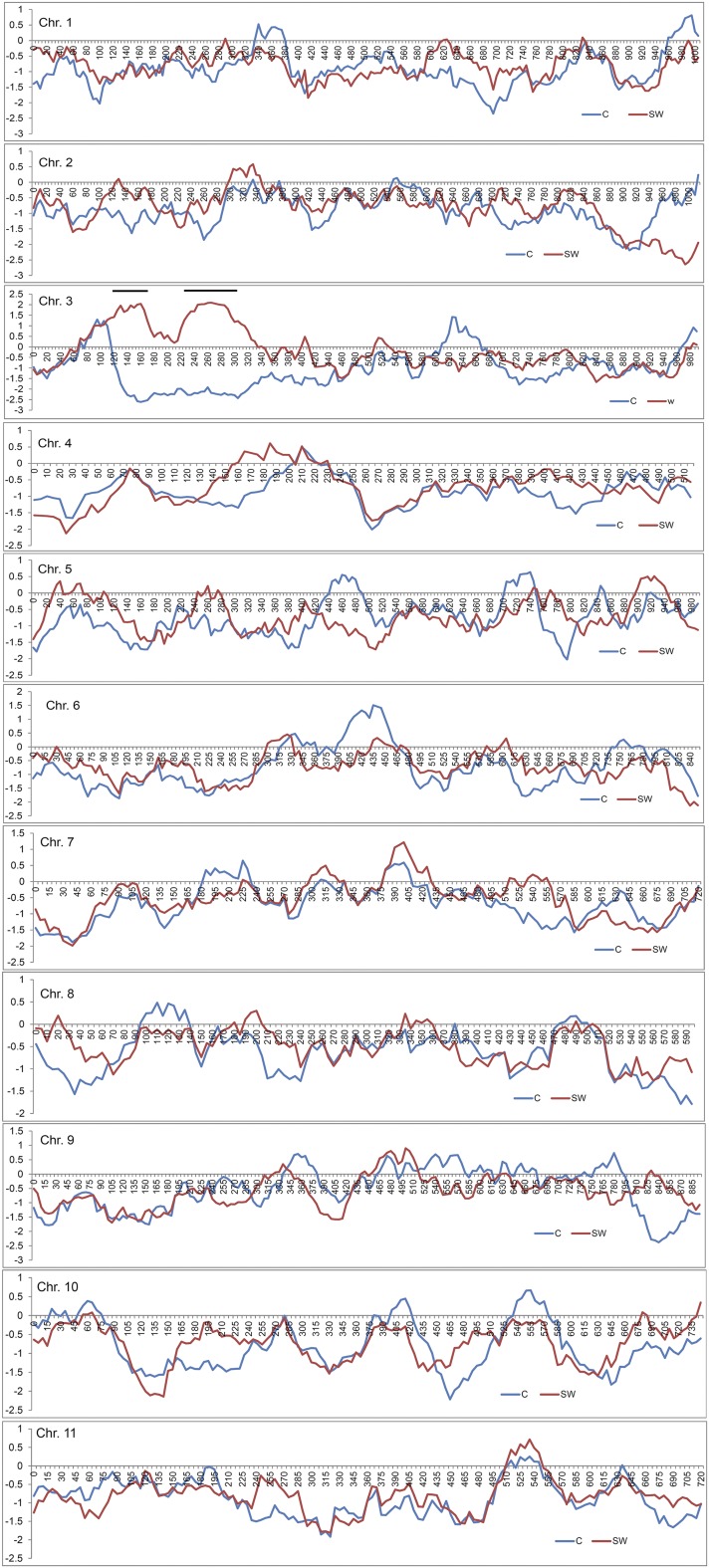
Genome-wide window-based Tajima’s *D* of cultivated (blue) and semi-wild watermelon (red) across various chromosomes. If Tajima’s D is negative for blue and positive for red, then that region of the genome is under selective sweep. Note two dark lines on chromosome 3 that showed strong signal for selective sweep.

We identified selection signatures across genomic regions in various chromosomes using Tajima’s *D*. Mean estimated Tajima’s *D* for every 100-kb window across the length of various chromosomes for sweet and semi-wild watermelon is provided in Figure 7. We identified a strong domestication signature on chromosome 3. We also estimated the observed and expected nucleotide diversity (π and θ) of semi-wild and cultivated watermelon, which suggested the narrowing of genetic diversity in sweet watermelon. The mean observed nucleotide diversity ranged from 0.163 ± 0.044 (chromosome 6) to 0.217 ± 0.072 (chromosome 3) for semi-wild accessions as compared with 0.147 ± 0.043 (chromosome 1) to 0.170 ± 0.046 (chromosome 9) for sweet watermelon accessions. Differences in both observed and expected nucleotide diversities for semi-wild compared with sweet watermelon on chromosome 3 contrast with those for the other chromosomes (Table 1).

## Discussion

### Genotyping by sequencing

Many of the challenges with complex crop genomes can be overcome by GBS (Glaubitz *et al.* 2014). This protocol is a multiplexed, high-throughput, and low-cost method to explore the genetic diversity in populations (Elshire *et al.* 2011). In this article, we report a robust set of 10,480 SNPs included in a high-density genetic map and 1563 SNPs in a diversity panel. Before this report, Sandlin *et al.* (2012) and Nimmakayala *et al.* (2014) developed 1073 and 384 SNPs, respectively, for watermelon and showed their use in genetic mapping and genetic diversity studies. Guo *et al.* (2013) resequenced 20 watermelon accessions comprising sweet, semi-wild, and wild watermelon to identify 6,784,860 candidate SNPs and 965,006 small insertions/deletions (indels).

### Use of high-density genetic maps in genomic research

Genotyping samples of a large population by sequencing presents some advantages over conventional genotyping methods. For example, GBS does not require previous characterization of polymorphisms for detection. Neves *et al.* (2013) showed that this advantage is of greater importance for a segregating population because even if previously characterized polymorphisms are available, the sites that will segregate in a single biparental segregating population and have potential to be mapped are unknown. Sub-centimorgan genetic maps such as that developed for watermelon in the current research provide a valuable resource for gene positioning on chromosomes and a guide for the assembly of a reference pine genome (Neves *et al.* 2013). High-density maps can contribute to a fundamental knowledge of genome structure and have numerous applications in breeding programs to enable genomic selection and precise mapping of agronomically important genes for marker-assisted selection (Hahn *et al.* 2014; Poland *et al.* 2012). Linkage maps are indispensable tools to study virtually every aspect of genome biology. Genomic features associated with the GWRR include GC content, gene density, gene expression, epigenetic modifications, nucleosome formation, repetitive element composition, isochore structure, and patterns of genetic variation and differentiation within and between populations (Tortereau *et al.* 2012). Thus, increasingly dense recombination maps have been constructed in the “post-genomic era” for species such as human and mouse, focusing on identifying hot spots of recombination and, recently, variation in the use of these hot spots between populations (Paigen and Petkov 2010).

In this study of the watermelon genome, we identified the most robust skeletal markers with strong linkages and high confidence levels. This skeletal map further allowed for incorporating hundreds of add-on SNPs and gave a picture of haplotypic diversity and genome structure across the populations. This high-density genetic map will be of use in correcting the existing reference genome sequence for watermelon and assembly of future whole-genome resequencing endeavors. Such an approach of contextually ordering the reference sequence assisted by GBS maps will, in turn, enable better SNP calling in future GBS datasets and haplotypic imputation of missing data (Poland *et al.* 2012; Tortereau *et al.* 2012). GBS methods have vastly improved the resolution and accuracy of genetic linkage maps by increasing both the number of marker loci and the number of individuals genotyped at these loci (Holeski *et al.* 2014). Our high-density recombination map of the watermelon genome has substantially higher resolution than previously published maps. A major goal is to characterize broad patterns and features of genomes, including linkage and recombination rate variation. This high resolution allowed us to reveal low and high GWRRs across the genome, for insight into the association of nucleotide diversity levels with high and low GWRRs. In addition to their use in checking the assembly of genome sequences, high-density maps can help in understanding the evolution of understudied genomes by analysis of recombination (Tortereau *et al.* 2012; Van Elferink *et al.* 2010; Vingborg *et al.* 2009; Wong *et al.* 2010).

### LD, recombination rate, and nucleotide diversity

We found extensive LD across all chromosomes of watermelon. LD must be characterized before association study. In addition to LD variation between subpopulations, LD can vary greatly across a genome, often as a result of variation in recombination rates (Marsden *et al.* 2014). Our knowledge of recombination rates and patterns in plants is far from comprehensive (Gaut *et al.* 2007). However, compelling evidence indicates a central role for recombination, via its effect on mutation and selection, in the evolution of plant genomes (Gaut *et al.* 2007). Much additional study of recombination in watermelon is needed to investigate these ideas further. Finally, one must test for evidence of population structure, which results in allele frequency differences between subpopulations. Unless controlled for, such population structure may cause spurious LD between unlinked markers, thus resulting in false associations and/or inflated true associations (Lewis and Knight 2012). In the current study, levels of nucleotide diversity varied significantly both within and between chromosomes. We observed lower diversity combined with low recombination rate on chromosome 3, which showed selective sweep and signals of domestication. We noted a trend of suppressed recombination resulting in reduced diversity within and across the chromosomes. Li *et al.* (2014) hypothesized that an increasing number of recombinations in genomic areas that have undergone selective sweeps might be an important aspect of breaking the current yield barriers in breeding.

### Location of selective sweep across the genome

Guo *et al.* (2013) studied selective sweep in the watermelon genome by scanning genetic diversity (π*_mucosospermus_*/π_sweet watermelon_) among six accessions of *C. lanatus* subsp. *mucosospermus* and 11 accessions of sweet watermelon to identify domestication signals. The authors identified 108 regions (7.78 Mb) containing 741 candidate genes under selective sweep across the genome. Guo *et al.* (2013) further characterized a large region on chromosome 3 (from ∼3.4 to ∼5.6 Mb) with the highest nucleotide divergence among subsp. *mucosospermus* accessions as compared with sweet watermelon. This region contained the genes for regulating carbohydrate use, sugar-mediated signaling, carbohydrate metabolism, response to sucrose stimulus, regulation of nitrogen-compound metabolism, cellular response to nitrogen starvation, and growth. However, the study involved small sample sizes, which can suggest bias due to narrow genetic diversity, limited population history, selection timing, phasing error, and false LD resolution (Granka *et al.* 2012; Qanbari *et al.* 2012; Tang *et al.* 2007). In addition, selective sweep reduces variability around a selected site: new mutations would gradually appear at low frequencies, eventually causing a frequency spectrum (Oleksyk *et al.* 2010). Alternatively, balanced selection maintains a high proportion of frequency polymorphisms, thereby shifting the spectrum to the intermediate frequencies (Oleksyk *et al.* 2010).

A shift in frequency spectrum would cause changes in the occurrence of ancestral and derived alleles, necessitating use of the Tajima’s *D* test, an approach that compares the mean pair-wise difference between sequences in a population sample (*π*) with the number of differences estimated by using the number of polymorphic sites (*s*). Tajima’s *D* is 0 for neutral variation, positive when an excess of rare polymorphism indicates positive selection, and negative with an excess of high-frequency variants, which indicates balanced selection (Tajima 1989). We used Tajima’s *D* test to confirm the highest signal of purifying selection on chromosome 3, which provides strong evidence for the genes with important roles in ripening, sugar-mediated signaling, and carbohydrate transport and fruit development, as being important for sweet watermelon domestication.

## Conclusions

This study provided a high-density genetic map of *C. lanatus* var. *lanatus* containing a set of 10,480 SNPs and further characterized genomic features such as GWRR, LD, and selective sweep across the genome. High-density maps can be used in breeding programs for genomic selection and precise mapping of agronomically important genes for marker-assisted selection. The extent of LD is a key factor in determining the number of markers needed for GWAS and genomic selection. Our research provides resources for association mapping to identify functional variation associated with important agronomic and economic traits in watermelon.

## Supplementary Material

Supporting Information
