# Supplementary material for: High-Resolution Genetic Map for Understanding the Effect of Genome-Wide Recombination Rate on Nucleotide Diversity in Watermelon
Source: G3 (Bethesda). 2014 Sep 15;4(11):2219–30. doi: 10.1534/g3.114.012815 (PMC4232547; doi:10.1534/g3.114.012815)
Supplement: Supporting Information [file supp_4_11_2219__index.html]

High-Resolution Genetic Map for Understanding the Effect of Genome-Wide Recombination Rate on Nucleotide Diversity in Watermelon — Supporting Information 

# High-Resolution Genetic Map for Understanding the Effect of Genome-Wide Recombination Rate on Nucleotide Diversity in Watermelon

## Supporting Information for Reddy *et al.*, 2014

**Files in this Data Supplement:**

- Supporting Information - Figures S1-S3 and Tables S1-S13 (PDF, 4 MB)
- Figure S1 - A and B: Chromosome-wise neighbor-joining trees for sweet, semi-wild and wild watermelon (Green: sweet, pink: semi-wild, blue: wild). (PDF, 382 KB)
- Figure S2 - ΔK distribution for various clusters estimated by use of Structure Harvester. (PDF, 179 KB)
- Figure S3 - 1 to 11: High-resolution genetic maps of various chromosomes consisting of add-on markers. (PDF, 3 MB)
- Table S1 - Details of various accessions used in the study. (.xlsx, 14 KB)
- Table S2 - Eigen values of first 2 vectors for various accessions used for principal component analysis. (.xlsx, 13 KB)
- Table S3 - Add-on markers mapped to chromosome 1. (.xls, 162 KB)
- Table S4 - Add-on markers mapped to chromosome 2. (.xls, 124 KB)
- Table S5 - Add-on markers mapped to chromosome 3. (.xls, 107 KB)
- Table S6 - Add-on markers mapped to chromosome 4. (.xls, 85 KB)
- Table S7 - Add-on markers mapped to chromosome 5. (.xls, 171 KB)
- Table S8 - Add-on markers mapped to chromosome 6. (.xls, 114 KB)
- Table S9 - Add-on markers mapped to chromosome 7. (.xls, 132 KB)
- Table S10 - Add-on markers mapped to chromosome 8. (.xls, 105 KB)
- Table S11 - Add-on markers mapped to chromosome 9. (.xls, 140 KB)
- Table S12 - Add-on markers mapped to chromosome 10. (.xls, 146 KB)
- Table S13 - Add-on markers mapped to chromosome 11. (.xls, 127 KB)
