# Supplementary material for: High-Resolution Genetic Map for Understanding the Effect of Genome-Wide Recombination Rate on Nucleotide Diversity in Watermelon
Source: G3 (Bethesda). 2014 Sep 15;4(11):2219–30. doi: 10.1534/g3.114.012815 (PMC4232547; doi:10.1534/g3.114.012815)
Supplement: Supporting Information [file supp_g3.114.012815_012815SI.pdf]

G3·Genes | Genomes | Genetics

High-Resolution Genetic Map for Understanding the Effect of Genome-Wide Recombination Rate on Nucleotide Diversity in Watermelon

Umesh K. Reddy [1, 5*], Padma Nimmakayala[1*], Amnon Levi[2], Venkata Lakshmi Abburi[1], Thangasamy Saminathan[1], Yan. R. Tomason[1], Gopinath Vajja[1], Rishi Reddy[1], Lavanya Abburi[1], Todd C. Wehner[3], Yefim Ronin[4] and Abraham Karol[4]

[1] Gus R. Douglass Institute, Department of Biology, West Virginia State University, WV 25112-1000, USA

[2]U.S. Vegetable Laboratory, USDA-ARS, 2875 Savannah Highway, Charleston, SC 29414, USA

[3]Department of Horticultural Science, North Carolina State University, Raleigh, NC 27695-7609, USA

[4]Institute of Evolution, Haifa University, Haifa 31905, Israel

[5]Author for correspondence: e-mail: ureddy@wvstateu.edu; Telephone: (304) 766 3066; Fax: 304-766-4199

*Authors contributed equally

DOI: 10.1534/g3.114.012815

A.

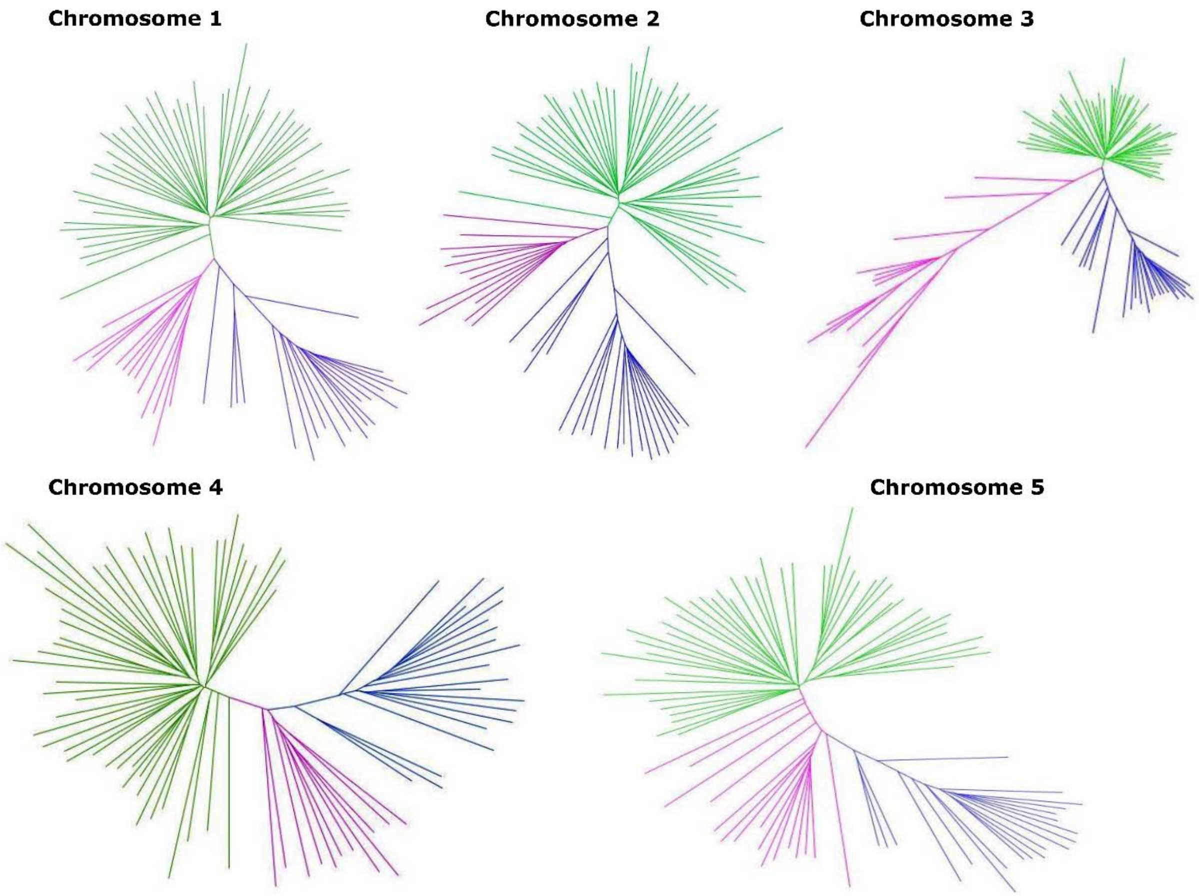
Chromosome 1
Chromosome 2
Chromosome 3
Chromosome 4
Chromosome 5

B.

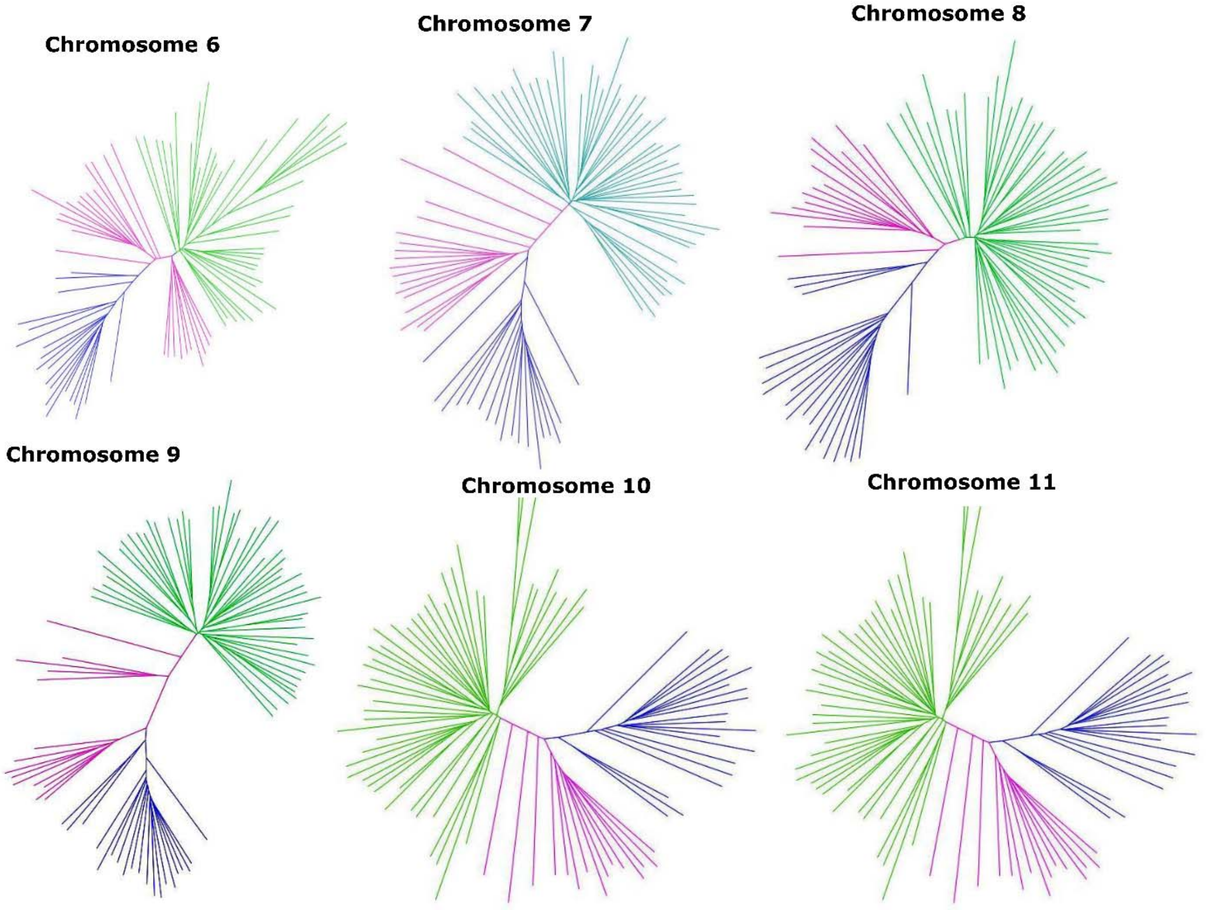

Figure S1 A and B: Chromosome-wise neighbor-joining trees for sweet, semi-wild and wild watermelon (Green: sweet, pink: semi-wild, blue: wild)

Burnin iterations = 100000
MCMC iterations = 500000
Replications = 4

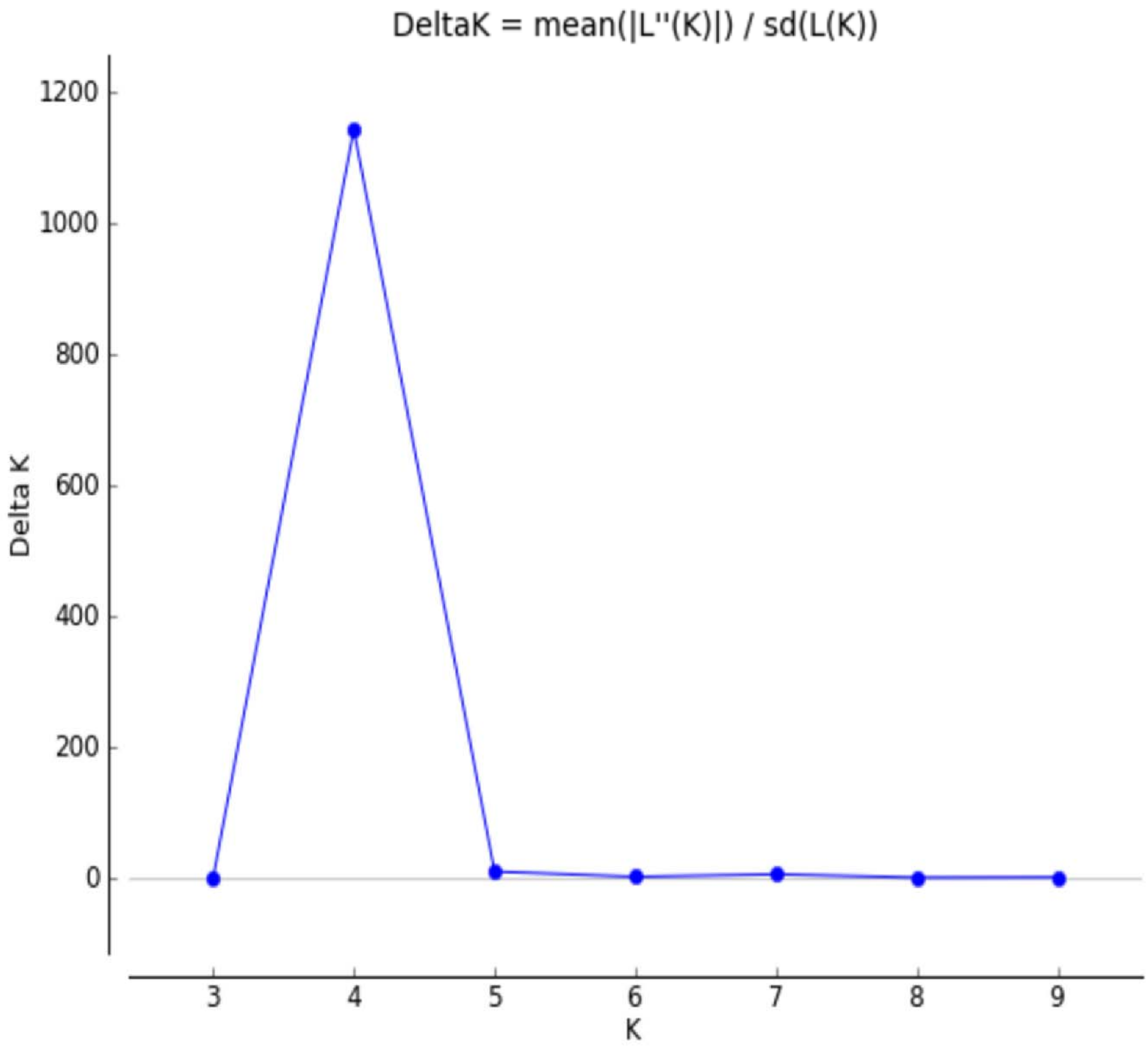

Figure S2 ΔK distribution for various clusters estimated by use of Structure Harvester. Highest ΔK value indicate the optimum cluster (K) for the current watermelon population.

Chr-1

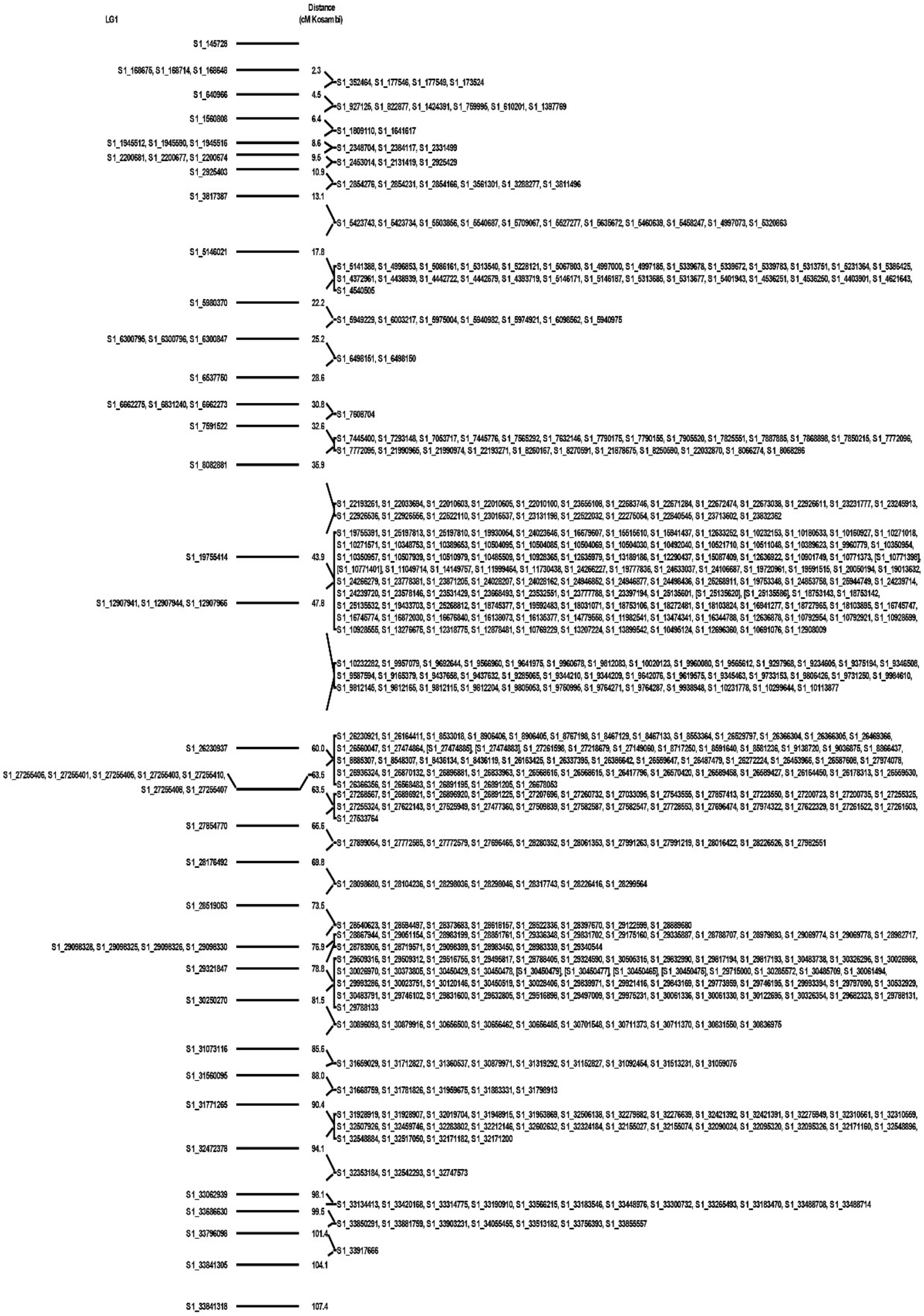
LG1
Distance (cM Kosambi)
S1_145728
S1_168675, S1_168714, S1_168648
2.3
S1_640966
4.5
S1_1560808
6.4
S1_1945512, S1_1945590, S1_1945516
8.6
S1_2200681, S1_2200677, S1_2200674
9.5
S1_2925403
10.9
S1_3817387
13.1
S1_5146021
17.8
S1_5980370
22.2
S1_6300795, S1_6300796, S1_6300847
25.2
S1_6537760
28.6
S1_6662275, S1_6831240, S1_6662273
30.8
S1_7591522
32.6
S1_8082881
35.9
S1_19755414
43.9
S1_12907941, S1_12907944, S1_12907966
47.8
S1_26230937
60.0
S1_27255406, S1_27255401, S1_27255405, S1_27255403, S1_27255410,
S1_27255408, S1_27255407
63.5
63.5
S1_27854770
66.6
S1_28176492
69.8
S1_28519053
73.5
S1_29098328, S1_29098325, S1_29098326, S1_29098330
76.9
S1_29321847
78.8
S1_30250270
81.5
S1_31073116
85.6
S1_31560095
88.0
S1_31771265
90.4
S1_32472378
94.1
S1_33062939
98.1
S1_33686630
99.5
S1_33796098
101.4
S1_33841305
104.1
S1_33841318
107.4

Chr-2

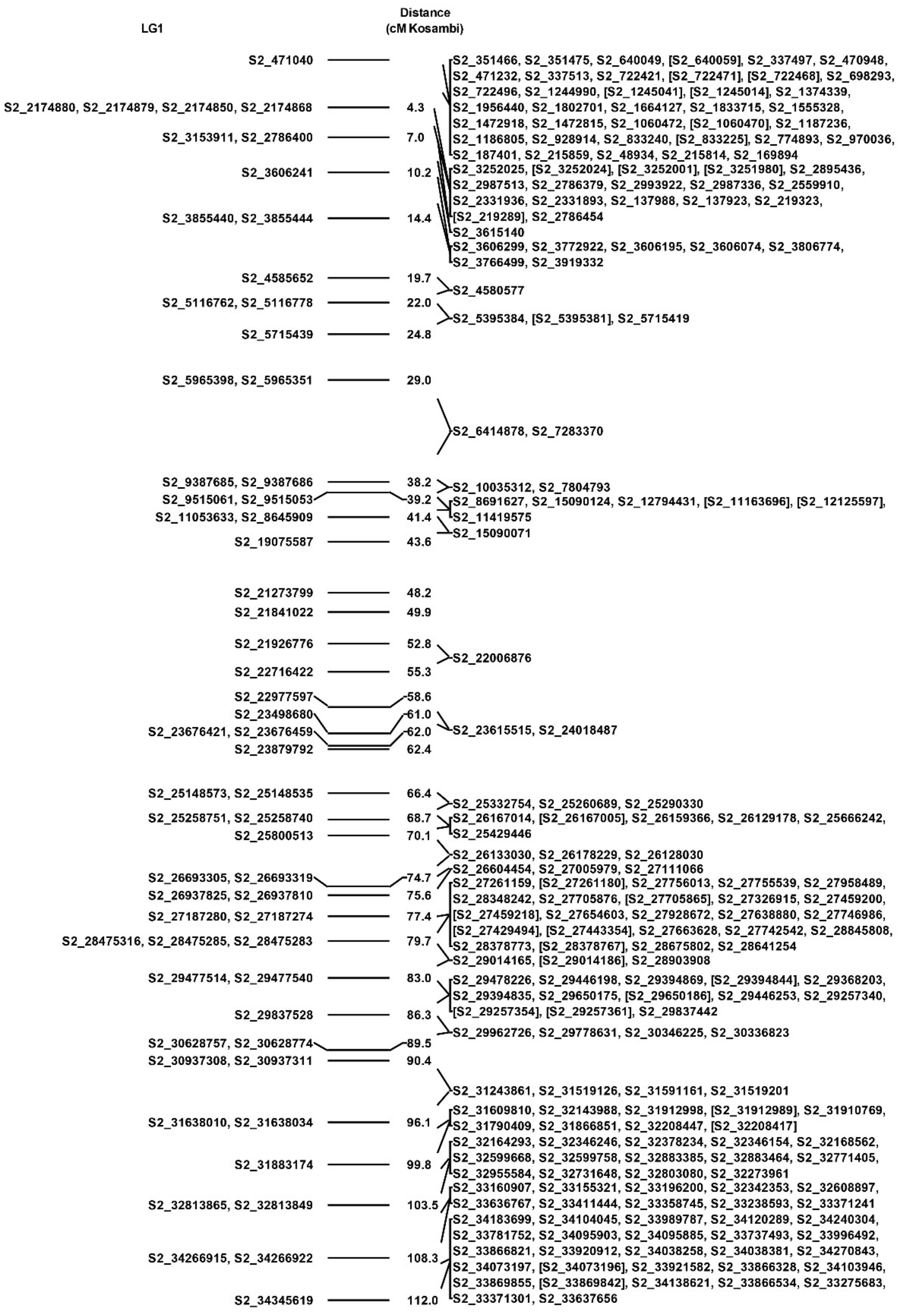
LG1
Distance
(cM Kosambi)
S2_471040
S2_2174880, S2_2174879, S2_2174850, S2_2174868 4.3
S2_3153911, S2_2786400 7.0
S2_3606241 10.2
S2_3855440, S2_3855444 14.4
S2_4585652 19.7
S2_5116762, S2_5116778 22.0
S2_5715439 24.8
S2_5965398, S2_5965351 29.0
S2_9387685, S2_9387686 38.2
S2_9515061, S2_9515053 39.2
S2_11053633, S2_8645909 41.4
S2_19075587 43.6
S2_21273799 48.2
S2_21841022 49.9
S2_21926776 52.8
S2_22716422 55.3
S2_22977597 58.6
S2_23498680 61.0
S2_23676421, S2_23676459 62.0
S2_23879792 62.4
S2_25148573, S2_25148535 66.4
S2_25258751, S2_25258740 68.7
S2_25800513 70.1
S2_26693305, S2_26693319 74.7
S2_26937825, S2_26937810 75.6
S2_27187280, S2_27187274 77.4
S2_28475316, S2_28475285, S2_28475283 79.7
S2_29477514, S2_29477540 83.0
S2_29837528 86.3
S2_30628757, S2_30628774 89.5
S2_30937308, S2_30937311 90.4
S2_31638010, S2_31638034 96.1
S2_31883174 99.8
S2_32813865, S2_32813849 103.5
S2_34266915, S2_34266922 108.3
S2_34345619 112.0
S2_351466, S2_351475, S2_640049, [S2_640059], S2_337497, S2_470948,
S2_471232, S2_337513, S2_722421, [S2_722471], [S2_722468], S2_698293,
S2_722496, S2_1244990, [S2_1245041], [S2_1245014], S2_1374339,
S2_1956440, S2_1802701, S2_1664127, S2_1833715, S2_1555328,
S2_1472918, S2_1472815, S2_1060472, [S2_1060470], S2_1187236,
S2_1186805, S2_928914, S2_833240, [S2_833225], S2_774893, S2_970036,
S2_187401, S2_215859, S2_48934, S2_215814, S2_169894
S2_3252025, [S2_3252024], [S2_3252001], [S2_3251980], S2_2895436,
S2_2987513, S2_2786379, S2_2993922, S2_2987336, S2_2559910,
S2_2331936, S2_2331893, S2_137988, S2_137923, S2_219323,
[S2_219289], S2_2786454
S2_3615140
S2_3606299, S2_3772922, S2_3606195, S2_3606074, S2_3806774,
S2_3766499, S2_3919332
S2_4580577
S2_5395384, [S2_5395381], S2_5715419
S2_6414878, S2_7283370
S2_10035312, S2_7804793
S2_8691627, S2_15090124, S2_12794431, [S2_11163696], [S2_12125597],
S2_11419575
S2_15090071
S2_22006876
S2_23615515, S2_24018487
S2_25332754, S2_25260689, S2_25290330
S2_26167014, [S2_26167005], S2_26159366, S2_26129178, S2_25666242,
S2_25429446
S2_26133030, S2_26178229, S2_26128030
S2_26604454, S2_27005979, S2_27111066
S2_27261159, [S2_27261180], S2_27756013, S2_27755539, S2_27958489,
S2_28348242, S2_27705876, [S2_27705865], S2_27326915, S2_27459200,
[S2_27459218], S2_27654603, S2_27928672, S2_27638880, S2_27746986,
[S2_27429494], [S2_27443354], S2_27663628, S2_27742542, S2_28845808,
S2_28378773, [S2_28378767], S2_28675802, S2_28641254
S2_29014165, [S2_29014186], S2_28903908
S2_29478226, S2_29446198, S2_29394869, [S2_29394844], S2_29368203,
S2_29394835, S2_29650175, [S2_29650186], S2_29446253, S2_29257340,
[S2_29257354], [S2_29257361], S2_29837442
S2_29962726, S2_29778631, S2_30346225, S2_30336823
S2_31243861, S2_31519126, S2_31591161, S2_31519201
S2_31609810, S2_32143988, S2_31912998, [S2_31912989], S2_31910769,
S2_31790409, S2_31866851, S2_32208447, [S2_32208417]
S2_32164293, S2_32346246, S2_32378234, S2_32346154, S2_32168562,
S2_32599668, S2_32599758, S2_32883385, S2_32883464, S2_32771405,
S2_32955584, S2_32731648, S2_32803080, S2_32273961
S2_33160907, S2_33155321, S2_33196200, S2_32342353, S2_32608897,
S2_33636767, S2_33411444, S2_33358745, S2_33238593, S2_33371241
S2_34183699, S2_34104045, S2_33989787, S2_34120289, S2_34240304,
S2_33781752, S2_34095903, S2_34095885, S2_33737493, S2_33996492,
S2_33866821, S2_33920912, S2_34038258, S2_34038381, S2_34270843,
S2_34073197, [S2_34073196], S2_33921582, S2_33866328, S2_34103946,
S2_33869855, [S2_33869842], S2_34138621, S2_33866534, S2_33275683,
S2_33371301, S2_33637656

Chr-3

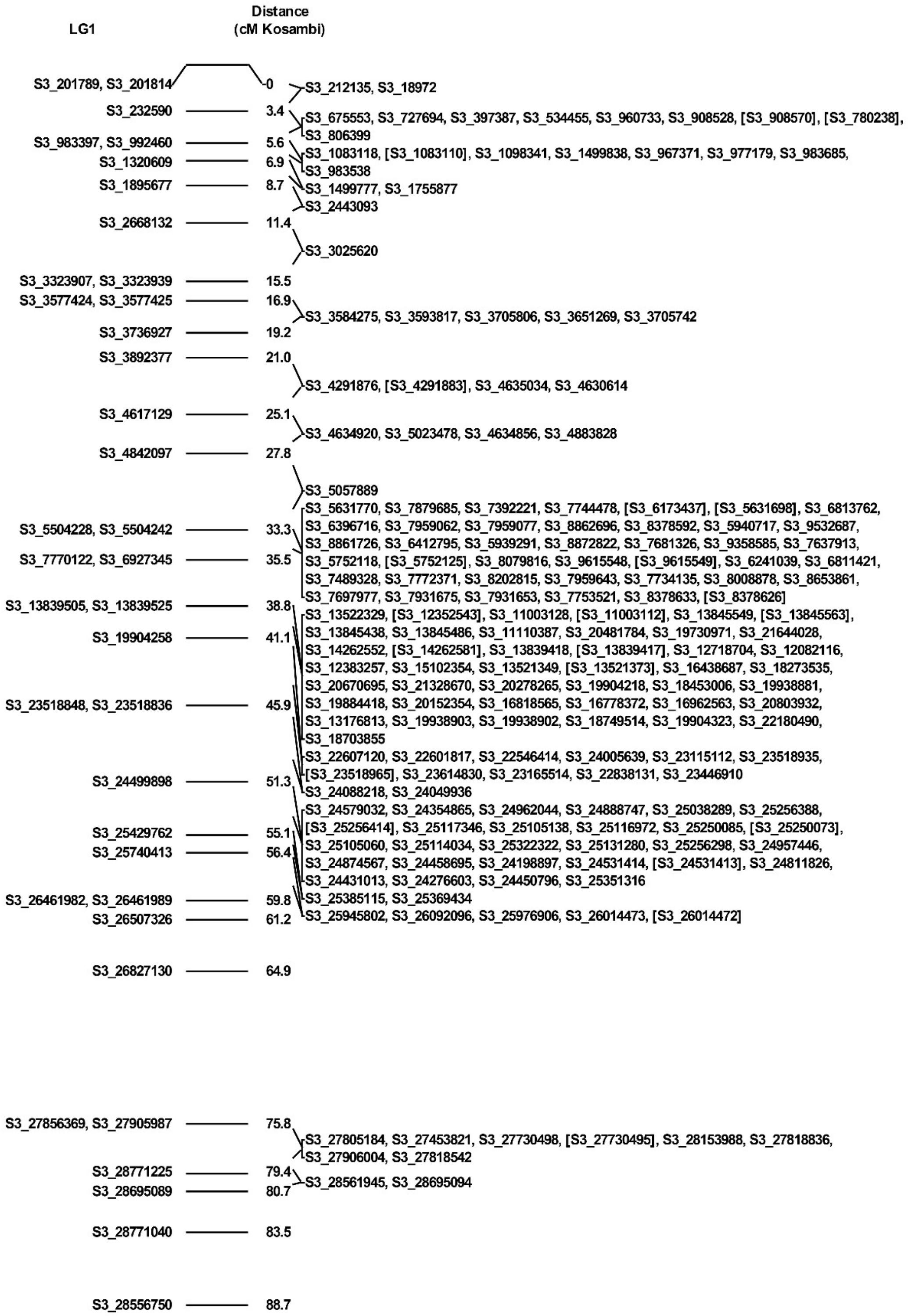
LG1
Distance
(cM Kosambi)
S3_201789, S3_201814
0
S3_212135, S3_18972
S3_232590
3.4
S3_675553, S3_727694, S3_397387, S3_534455, S3_960733, S3_908528, [S3_908570], [S3_780238],
S3_806399
S3_983397, S3_992460
5.6
S3_1083118, [S3_1083110], S3_1098341, S3_1499838, S3_967371, S3_977179, S3_983685,
S3_983538
S3_1320609
6.9
S3_1895677
8.7
S3_1499777, S3_1755877
S3_2443093
S3_2668132
11.4
S3_3025620
S3_3323907, S3_3323939
15.5
S3_3577424, S3_3577425
16.9
S3_3584275, S3_3593817, S3_3705806, S3_3651269, S3_3705742
S3_3736927
19.2
S3_3892377
21.0
S3_4291876, [S3_4291883], S3_4635034, S3_4630614
S3_4617129
25.1
S3_4634920, S3_5023478, S3_4634856, S3_4883828
S3_4842097
27.8
S3_5057889
S3_5631770, S3_7879685, S3_7392221, S3_7744478, [S3_6173437], [S3_5631698], S3_6813762,
S3_5504228, S3_5504242
33.3
S3_6396716, S3_7959062, S3_7959077, S3_8862696, S3_8378592, S3_5940717, S3_9532687,
S3_8861726, S3_6412795, S3_5939291, S3_8872822, S3_7681326, S3_9358585, S3_7637913,
S3_7770122, S3_6927345
35.5
S3_5752118, [S3_5752125], S3_8079816, S3_9615548, [S3_9615549], S3_6241039, S3_6811421,
S3_7489328, S3_7772371, S3_8202815, S3_7959643, S3_7734135, S3_8008878, S3_8653861,
S3_7697977, S3_7931675, S3_7931653, S3_7753521, S3_8378633, [S3_8378626]
S3_13839505, S3_13839525
38.8
S3_13522329, [S3_12352543], S3_11003128, [S3_11003112], S3_13845549, [S3_13845563],
S3_19904258
41.1
S3_13845438, S3_13845486, S3_11110387, S3_20481784, S3_19730971, S3_21644028,
S3_14262552, [S3_14262581], S3_13839418, [S3_13839417], S3_12718704, S3_12082116,
S3_12383257, S3_15102354, S3_13521349, [S3_13521373], S3_16438687, S3_18273535,
S3_20670695, S3_21328670, S3_20278265, S3_19904218, S3_18453006, S3_19938881,
S3_23518848, S3_23518836
45.9
S3_19884418, S3_20152354, S3_16818565, S3_16778372, S3_16962563, S3_20803932,
S3_13176813, S3_19938903, S3_19938902, S3_18749514, S3_19904323, S3_22180490,
S3_18703855
S3_22607120, S3_22601817, S3_22546414, S3_24005639, S3_23115112, S3_23518935,
[S3_23518965], S3_23614830, S3_23165514, S3_22838131, S3_23446910
S3_24499898
51.3
S3_24088218, S3_24049936
S3_24579032, S3_24354865, S3_24962044, S3_24888747, S3_25038289, S3_25256388,
S3_25429762
55.1
[S3_25256414], S3_25117346, S3_25105138, S3_25116972, S3_25250085, [S3_25250073],
S3_25740413
56.4
S3_25105060, S3_25114034, S3_25322322, S3_25131280, S3_25256298, S3_24957446,
S3_24874567, S3_24458695, S3_24198897, S3_24531414, [S3_24531413], S3_24811826,
S3_24431013, S3_24276603, S3_24450796, S3_25351316
S3_26461982, S3_26461989
59.8
S3_25385115, S3_25369434
S3_26507326
61.2
S3_25945802, S3_26092096, S3_25976906, S3_26014473, [S3_26014472]
S3_26827130
64.9
S3_27856369, S3_27905987
75.8
S3_27805184, S3_27453821, S3_27730498, [S3_27730495], S3_28153988, S3_27818836,
S3_27906004, S3_27818542
S3_28771225
79.4
S3_28561945, S3_28695094
S3_28695089
80.7
S3_28771040
83.5
S3_28556750
88.7

Chr-4

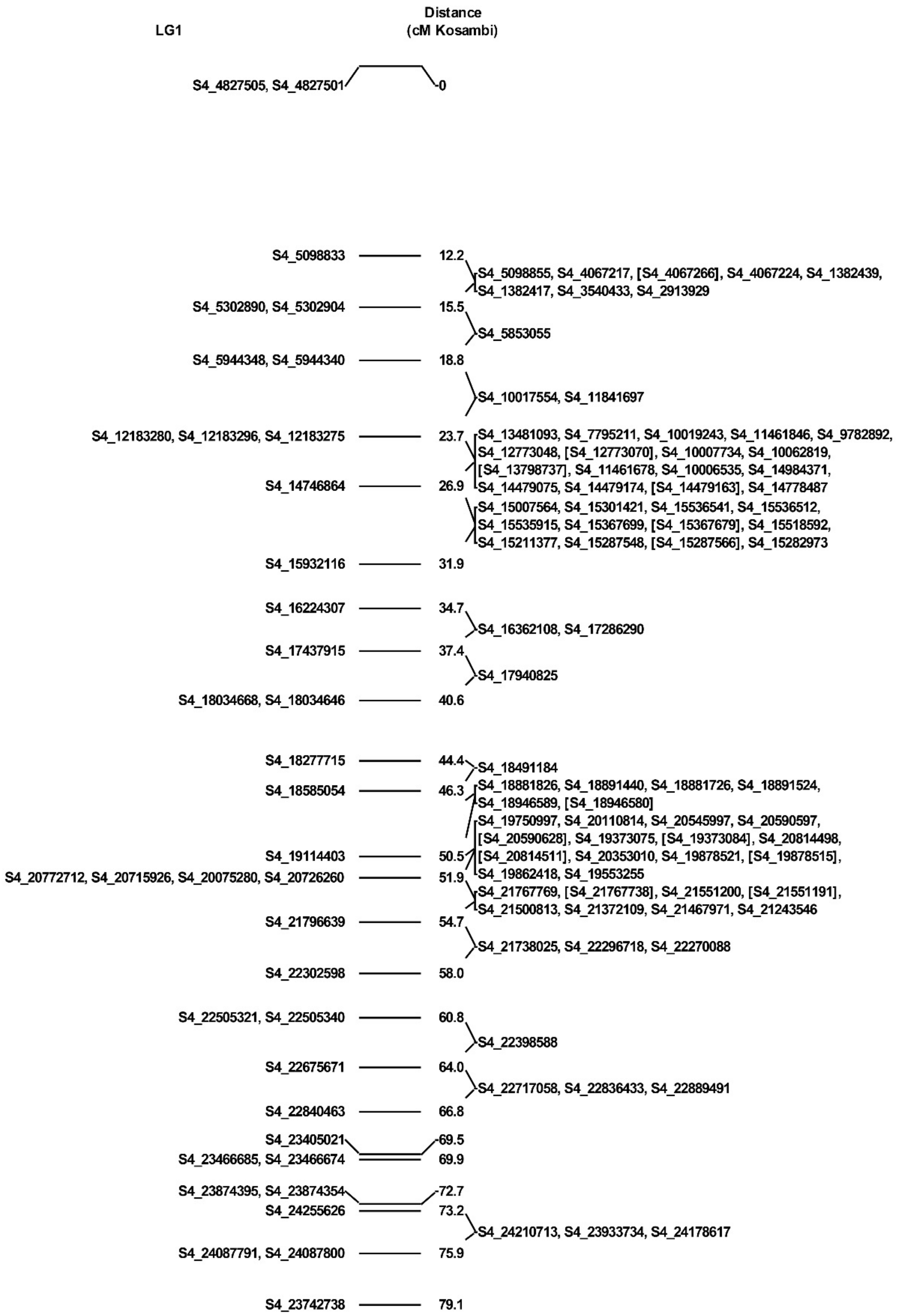

LG1
Distance
(cM Kosambi)
S4_4827505, S4_4827501
0
S4_5098833
12.2
S4_5098855, S4_4067217, [S4_4067266], S4_4067224, S4_1382439,
S4_1382417, S4_3540433, S4_2913929
S4_5302890, S4_5302904
15.5
S4_5853055
S4_5944348, S4_5944340
18.8
S4_10017554, S4_11841697
S4_12183280, S4_12183296, S4_12183275
23.7
S4_13481093, S4_7795211, S4_10019243, S4_11461846, S4_9782892,
S4_12773048, [S4_12773070], S4_10007734, S4_10062819,
[S4_13798737], S4_11461678, S4_10006535, S4_14984371,
S4_14746864
26.9
S4_14479075, S4_14479174, [S4_14479163], S4_14778487
S4_15007564, S4_15301421, S4_15536541, S4_15536512,
S4_15535915, S4_15367699, [S4_15367679], S4_15518592,
S4_15211377, S4_15287548, [S4_15287566], S4_15282973
S4_15932116
31.9
S4_16224307
34.7
S4_16362108, S4_17286290
S4_17437915
37.4
S4_17940825
S4_18034668, S4_18034646
40.6
S4_18277715
44.4
S4_18491184
S4_18585054
46.3
S4_18881826, S4_18891440, S4_18881726, S4_18891524,
S4_18946589, [S4_18946580]
S4_19750997, S4_20110814, S4_20545997, S4_20590597,
[S4_20590628], S4_19373075, [S4_19373084], S4_20814498,
S4_19114403
50.5
[S4_20814511], S4_20353010, S4_19878521, [S4_19878515],
S4_20772712, S4_20715926, S4_20075280, S4_20726260
51.9
S4_19862418, S4_19553255
S4_21767769, [S4_21767738], S4_21551200, [S4_21551191],
S4_21500813, S4_21372109, S4_21467971, S4_21243546
S4_21796639
54.7
S4_21738025, S4_22296718, S4_22270088
S4_22302598
58.0
S4_22505321, S4_22505340
60.8
S4_22398588
S4_22675671
64.0
S4_22717058, S4_22836433, S4_22889491
S4_22840463
66.8
S4_23405021
69.5
S4_23466685, S4_23466674
69.9
S4_23874395, S4_23874354
72.7
S4_24255626
73.2
S4_24210713, S4_23933734, S4_24178617
S4_24087791, S4_24087800
75.9
S4_23742738
79.1

Chr-5

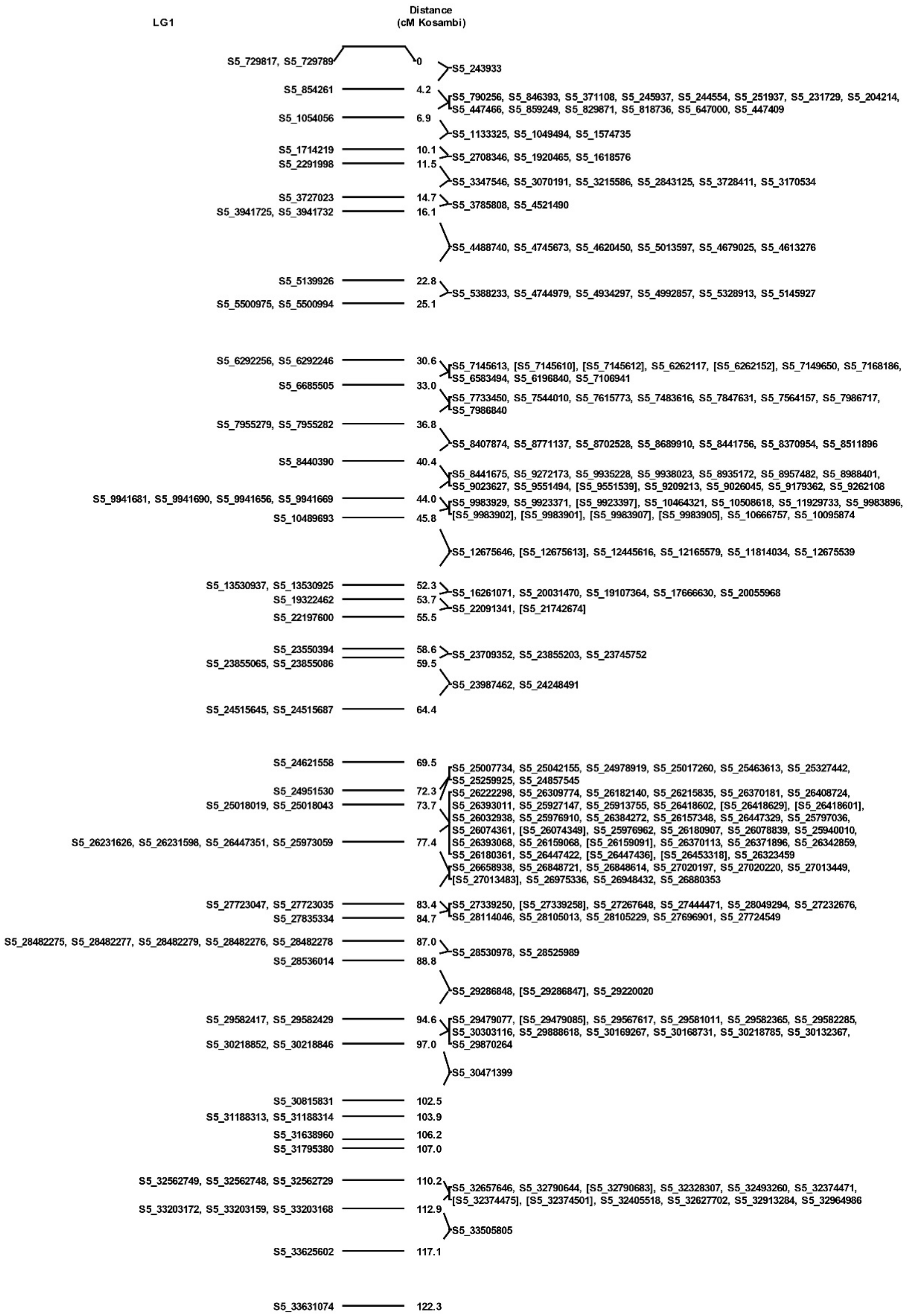
LG1
Distance
(cM Kosambi)
S5_729817, S5_729789 0
S5_243933
S5_854261 4.2
S5_790256, S5_846393, S5_371108, S5_245937, S5_244554, S5_251937, S5_231729, S5_204214,
S5_447466, S5_859249, S5_829871, S5_818736, S5_647000, S5_447409
S5_1054056 6.9
S5_1133325, S5_1049494, S5_1574735
S5_1714219 10.1
S5_2708346, S5_1920465, S5_1618576
S5_2291998 11.5
S5_3347546, S5_3070191, S5_3215586, S5_2843125, S5_3728411, S5_3170534
S5_3727023 14.7
S5_3785808, S5_4521490
S5_3941725, S5_3941732 16.1
S5_4488740, S5_4745673, S5_4620450, S5_5013597, S5_4679025, S5_4613276
S5_5139926 22.8
S5_5388233, S5_4744979, S5_4934297, S5_4992857, S5_5328913, S5_5145927
S5_5500975, S5_5500994 25.1
S5_6292256, S5_6292246 30.6
S5_7145613, [S5_7145610], [S5_7145612], S5_6262117, [S5_6262152], S5_7149650, S5_7168186,
S5_6583494, S5_6196840, S5_7106941
S5_6685505 33.0
S5_7733450, S5_7544010, S5_7615773, S5_7483616, S5_7847631, S5_7564157, S5_7986717,
S5_7986840
S5_7955279, S5_7955282 36.8
S5_8407874, S5_8771137, S5_8702528, S5_8689910, S5_8441756, S5_8370954, S5_8511896
S5_8440390 40.4
S5_8441675, S5_9272173, S5_9935228, S5_9938023, S5_8935172, S5_8957482, S5_8988401,
S5_9023627, S5_9551494, [S5_9551539], S5_9209213, S5_9026045, S5_9179362, S5_9262108
S5_9941681, S5_9941690, S5_9941656, S5_9941669 44.0
S5_9983929, S5_9923371, [S5_9923397], S5_10464321, S5_10508618, S5_11929733, S5_9983896,
[S5_9983902], [S5_9983901], [S5_9983907], [S5_9983905], S5_10666757, S5_10095874
S5_10489693 45.8
S5_12675646, [S5_12675613], S5_12445616, S5_12165579, S5_11814034, S5_12675539
S5_13530937, S5_13530925 52.3
S5_16261071, S5_20031470, S5_19107364, S5_17666630, S5_20055968
S5_19322462 53.7
S5_22091341, [S5_21742674]
S5_22197600 55.5
S5_23550394 58.6
S5_23709352, S5_23855203, S5_23745752
S5_23855065, S5_23855086 59.5
S5_23987462, S5_24248491
S5_24515645, S5_24515687 64.4
S5_24621558 69.5
S5_25007734, S5_25042155, S5_24978919, S5_25017260, S5_25463613, S5_25327442,
S5_25259925, S5_24857545
S5_24951530 72.3
S5_26222298, S5_26309774, S5_26182140, S5_26215835, S5_26370181, S5_26408724,
S5_25018019, S5_25018043 73.7
S5_26393011, S5_25927147, S5_25913755, S5_26418602, [S5_26418629], [S5_26418601],
S5_26032938, S5_25976910, S5_26384272, S5_26157348, S5_26447329, S5_25797036,
S5_26074361, [S5_26074349], S5_25976962, S5_26180907, S5_26078839, S5_25940010,
S5_26231626, S5_26231598, S5_26447351, S5_25973059 77.4
S5_26393068, S5_26159068, [S5_26159091], S5_26370113, S5_26371896, S5_26342859,
S5_26180361, S5_26447422, [S5_26447436], [S5_26453318], S5_26323459
S5_26658938, S5_26848721, S5_26848614, S5_27020197, S5_27020220, S5_27013449,
[S5_27013483], S5_26975336, S5_26948432, S5_26880353
S5_27723047, S5_27723035 83.4
S5_27339250, [S5_27339258], S5_27267648, S5_27444471, S5_28049294, S5_27232676,
S5_27835334 84.7
S5_28114046, S5_28105013, S5_28105229, S5_27696901, S5_27724549
S5_28482275, S5_28482277, S5_28482279, S5_28482276, S5_28482278 87.0
S5_28530978, S5_28525989
S5_28536014 88.8
S5_29286848, [S5_29286847], S5_29220020
S5_29582417, S5_29582429 94.6
S5_29479077, [S5_29479085], S5_29567617, S5_29581011, S5_29582365, S5_29582285,
S5_30303116, S5_29888618, S5_30169267, S5_30168731, S5_30218785, S5_30132367,
S5_30218852, S5_30218846 97.0
S5_29870264
S5_30471399
S5_30815831 102.5
S5_31188313, S5_31188314 103.9
S5_31638960 106.2
S5_31795380 107.0
S5_32562749, S5_32562748, S5_32562729 110.2
S5_32657646, S5_32790644, [S5_32790683], S5_32328307, S5_32493260, S5_32374471,
[S5_32374475], [S5_32374501], S5_32405518, S5_32627702, S5_32913284, S5_32964986
S5_33203172, S5_33203159, S5_33203168 112.9
S5_33505805
S5_33625602 117.1
S5_33631074 122.3

Chr-6

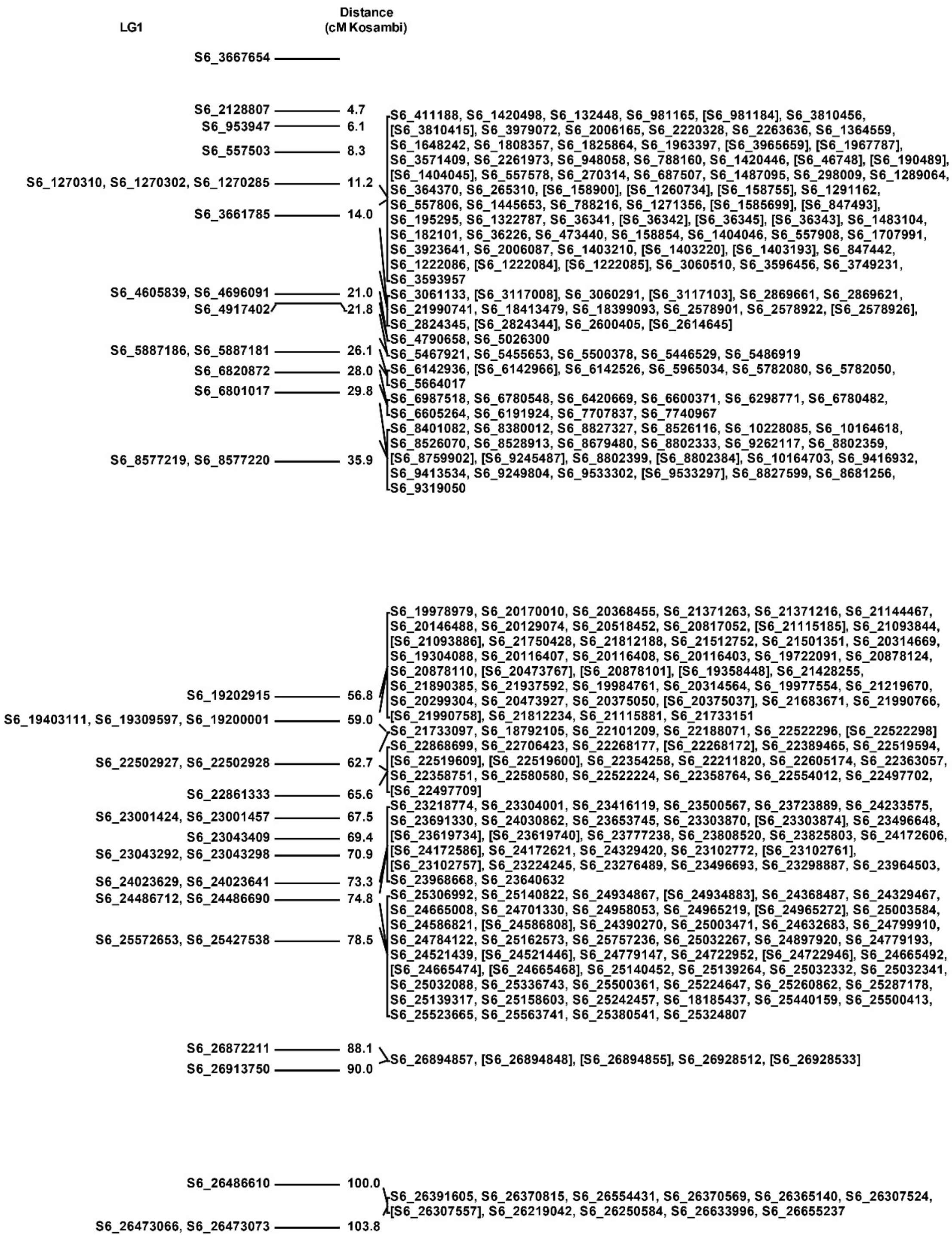
LG1
Distance (cM Kosambi)
S6_3667654
S6_2128807 4.7
S6_953947 6.1
S6_557503 8.3
S6_1270310, S6_1270302, S6_1270285 11.2
S6_3661785 14.0
S6_4605839, S6_4696091 21.0
S6_4917402 21.8
S6_5887186, S6_5887181 26.1
S6_6820872 28.0
S6_6801017 29.8
S6_8577219, S6_8577220 35.9
S6_411188, S6_1420498, S6_132448, S6_981165, [S6_981184], S6_3810456,
[S6_3810415], S6_3979072, S6_2006165, S6_2220328, S6_2263636, S6_1364559,
S6_1648242, S6_1808357, S6_1825864, S6_1963397, [S6_3965659], [S6_1967787],
S6_3571409, S6_2261973, S6_948058, S6_788160, S6_1420446, [S6_46748], [S6_190489],
[S6_1404045], S6_557578, S6_270314, S6_687507, S6_1487095, S6_298009, S6_1289064,
S6_364370, S6_265310, [S6_158900], [S6_1260734], [S6_158755], S6_1291162,
S6_557806, S6_1445653, S6_788216, S6_1271356, [S6_1585699], [S6_847493],
S6_195295, S6_1322787, S6_36341, [S6_36342], [S6_36345], [S6_36343], S6_1483104,
S6_182101, S6_36226, S6_473440, S6_158854, S6_1404046, S6_557908, S6_1707991,
S6_3923641, S6_2006087, S6_1403210, [S6_1403220], [S6_1403193], S6_847442,
S6_1222086, [S6_1222084], [S6_1222085], S6_3060510, S6_3596456, S6_3749231,
S6_3593957
S6_3061133, [S6_3117008], S6_3060291, [S6_3117103], S6_2869661, S6_2869621,
S6_21990741, S6_18413479, S6_18399093, S6_2578901, S6_2578922, [S6_2578926],
S6_2824345, [S6_2824344], S6_2600405, [S6_2614645]
S6_4790658, S6_5026300
S6_5467921, S6_5455653, S6_5500378, S6_5446529, S6_5486919
S6_6142936, [S6_6142966], S6_6142526, S6_5965034, S6_5782080, S6_5782050,
S6_5664017
S6_6987518, S6_6780548, S6_6420669, S6_6600371, S6_6298771, S6_6780482,
S6_6605264, S6_6191924, S6_7707837, S6_7740967
S6_8401082, S6_8380012, S6_8827327, S6_8526116, S6_10228085, S6_10164618,
S6_8526070, S6_8528913, S6_8679480, S6_8802333, S6_9262117, S6_8802359,
[S6_8759902], [S6_9245487], S6_8802399, [S6_8802384], S6_10164703, S6_9416932,
S6_9413534, S6_9249804, S6_9533302, [S6_9533297], S6_8827599, S6_8681256,
S6_9319050
S6_19202915 56.8
S6_19403111, S6_19309597, S6_19200001 59.0
S6_22502927, S6_22502928 62.7
S6_22861333 65.6
S6_23001424, S6_23001457 67.5
S6_23043409 69.4
S6_23043292, S6_23043298 70.9
S6_24023629, S6_24023641 73.3
S6_24486712, S6_24486690 74.8
S6_25572653, S6_25427538 78.5
S6_19978979, S6_20170010, S6_20368455, S6_21371263, S6_21371216, S6_21144467,
S6_20146488, S6_20129074, S6_20518452, S6_20817052, [S6_21115185], S6_21093844,
[S6_21093886], S6_21750428, S6_21812188, S6_21512752, S6_21501351, S6_20314669,
S6_19304088, S6_20116407, S6_20116408, S6_20116403, S6_19722091, S6_20878124,
S6_20878110, [S6_20473767], [S6_20878101], [S6_19358448], S6_21428255,
S6_21890385, S6_21937592, S6_19984761, S6_20314564, S6_19977554, S6_21219670,
S6_20299304, S6_20473927, S6_20375050, [S6_20375037], S6_21683671, S6_21990766,
[S6_21990758], S6_21812234, S6_21115881, S6_21733151
S6_21733097, S6_18792105, S6_22101209, S6_22188071, S6_22522296, [S6_22522298]
S6_22868699, S6_22706423, S6_22268177, [S6_22268172], S6_22389465, S6_22519594,
[S6_22519609], [S6_22519600], S6_22354258, S6_22211820, S6_22605174, S6_22363057,
S6_22358751, S6_22580580, S6_22522224, S6_22358764, S6_22554012, S6_22497702,
[S6_22497709]
S6_23218774, S6_23304001, S6_23416119, S6_23500567, S6_23723889, S6_24233575,
S6_23691330, S6_24030862, S6_23653745, S6_23303870, [S6_23303874], S6_23496648,
[S6_23619734], [S6_23619740], S6_23777238, S6_23808520, S6_23825803, S6_24172606,
[S6_24172586], S6_24172621, S6_24329420, S6_23102772, [S6_23102761],
[S6_23102757], S6_23224245, S6_23276489, S6_23496693, S6_23298887, S6_23964503,
S6_23968668, S6_23640632
S6_25306992, S6_25140822, S6_24934867, [S6_24934883], S6_24368487, S6_24329467,
S6_24665008, S6_24701330, S6_24958053, S6_24965219, [S6_24965272], S6_25003584,
S6_24586821, [S6_24586808], S6_24390270, S6_25003471, S6_24632683, S6_24799910,
S6_24784122, S6_25162573, S6_25757236, S6_25032267, S6_24897920, S6_24779193,
S6_24521439, [S6_24521446], S6_24779147, S6_24722952, [S6_24722946], S6_24665492,
[S6_24665474], [S6_24665468], S6_25140452, S6_25139264, S6_25032332, S6_25032341,
S6_25032088, S6_25336743, S6_25500361, S6_25224647, S6_25260862, S6_25287178,
S6_25139317, S6_25158603, S6_25242457, S6_18185437, S6_25440159, S6_25500413,
S6_25523665, S6_25563741, S6_25380541, S6_25324807
S6_26872211 88.1
S6_26913750 90.0
S6_26894857, [S6_26894848], [S6_26894855], S6_26928512, [S6_26928533]
S6_26486610 100.0
S6_26473066, S6_26473073 103.8
S6_26391605, S6_26370815, S6_26554431, S6_26370569, S6_26365140, S6_26307524,
[S6_26307557], S6_26219042, S6_26250584, S6_26633996, S6_26655237

Chr-7

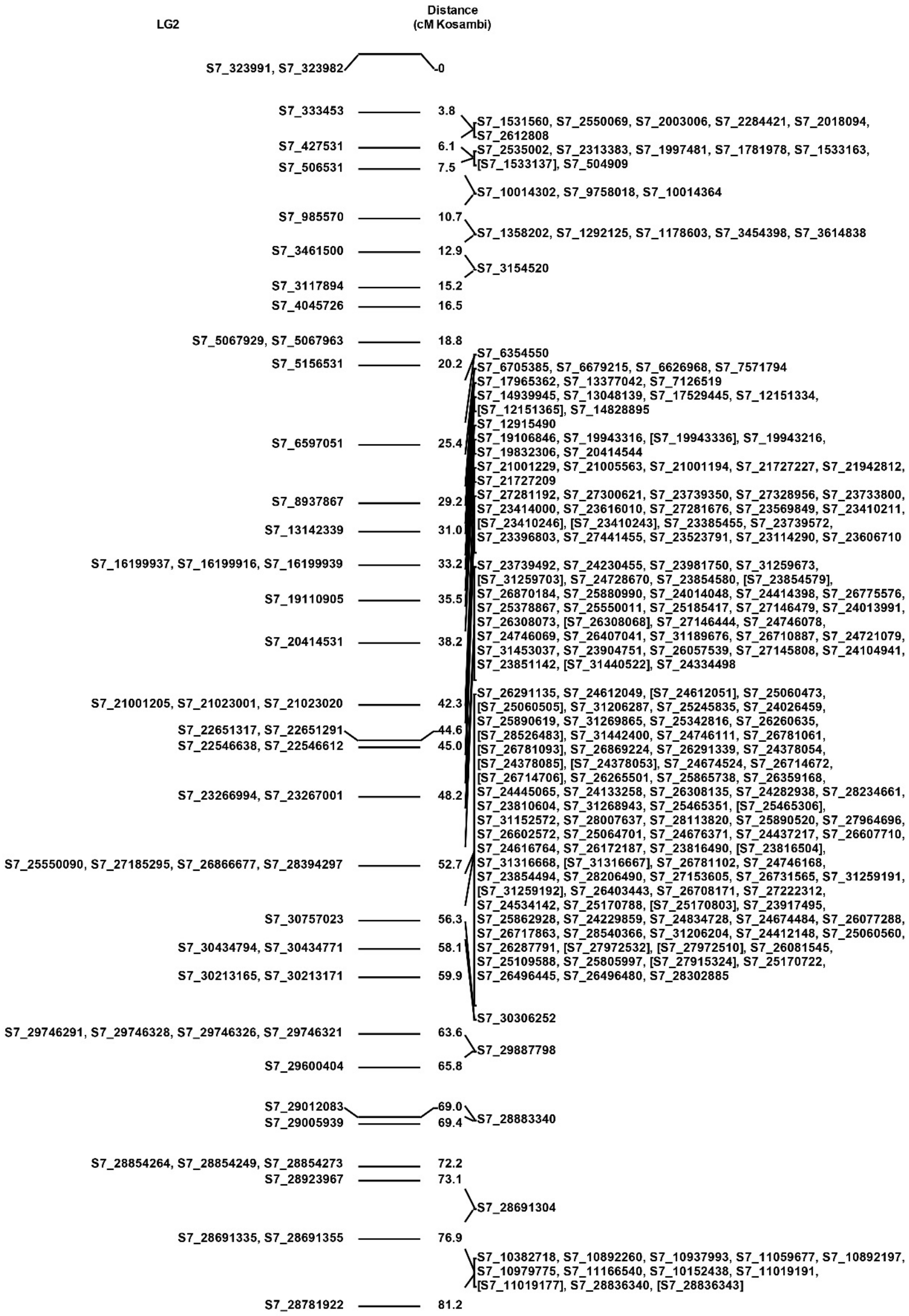

LG2
Distance (cM Kosambi)
S7_323991, S7_323982 0
S7_333453 3.8
S7_427531 6.1
S7_506531 7.5
S7_985570 10.7
S7_3461500 12.9
S7_3117894 15.2
S7_4045726 16.5
S7_5067929, S7_5067963 18.8
S7_5156531 20.2
S7_6597051 25.4
S7_8937867 29.2
S7_13142339 31.0
S7_16199937, S7_16199916, S7_16199939 33.2
S7_19110905 35.5
S7_20414531 38.2
S7_21001205, S7_21023001, S7_21023020 42.3
S7_22651317, S7_22651291 44.6
S7_22546638, S7_22546612 45.0
S7_23266994, S7_23267001 48.2
S7_25550090, S7_27185295, S7_26866677, S7_28394297 52.7
S7_30757023 56.3
S7_30434794, S7_30434771 58.1
S7_30213165, S7_30213171 59.9
S7_29746291, S7_29746328, S7_29746326, S7_29746321 63.6
S7_29600404 65.8
S7_29012083 69.0
S7_29005939 69.4
S7_28854264, S7_28854249, S7_28854273 72.2
S7_28923967 73.1
S7_28691335, S7_28691355 76.9
S7_28781922 81.2
S7_1531560, S7_2550069, S7_2003006, S7_2284421, S7_2018094, S7_2612808
S7_2535002, S7_2313383, S7_1997481, S7_1781978, S7_1533163, [S7_1533137], S7_504909
S7_10014302, S7_9758018, S7_10014364
S7_1358202, S7_1292125, S7_1178603, S7_3454398, S7_3614838
S7_3154520
S7_6354550
S7_6705385, S7_6679215, S7_6626968, S7_7571794
S7_17965362, S7_13377042, S7_7126519
S7_14939945, S7_13048139, S7_17529445, S7_12151334, [S7_12151365], S7_14828895
S7_12915490
S7_19106846, S7_19943316, [S7_19943336], S7_19943216, S7_19832306, S7_20414544
S7_21001229, S7_21005563, S7_21001194, S7_21727227, S7_21942812, S7_21727209
S7_27281192, S7_27300621, S7_23739350, S7_27328956, S7_23733800, S7_23414000, S7_23616010, S7_27281676, S7_23569849, S7_23410211, [S7_23410246], [S7_23410243], S7_23385455, S7_23739572, S7_23396803, S7_27441455, S7_23523791, S7_23114290, S7_23606710
S7_23739492, S7_24230455, S7_23981750, S7_31259673, [S7_31259703], S7_24728670, S7_23854580, [S7_23854579], S7_26870184, S7_25880990, S7_24014048, S7_24414398, S7_26775576, S7_25378867, S7_25550011, S7_25185417, S7_27146479, S7_24013991, S7_26308073, [S7_26308068], S7_27146444, S7_24746078, S7_24746069, S7_26407041, S7_31189676, S7_26710887, S7_24721079, S7_31453037, S7_23904751, S7_26057539, S7_27145808, S7_24104941, S7_23851142, [S7_31440522], S7_24334498
S7_26291135, S7_24612049, [S7_24612051], S7_25060473, [S7_25060505], S7_31206287, S7_25245835, S7_24026459, S7_25890619, S7_31269865, S7_25342816, S7_26260635, [S7_28526483], S7_31442400, S7_24746111, S7_26781061, [S7_26781093], S7_26869224, S7_26291339, S7_24378054, [S7_24378085], [S7_24378053], S7_24674524, S7_26714672, [S7_26714706], S7_26265501, S7_25865738, S7_26359168, S7_24445065, S7_24133258, S7_26308135, S7_24282938, S7_28234661, S7_23810604, S7_31268943, S7_25465351, [S7_25465306], S7_31152572, S7_28007637, S7_28113820, S7_25890520, S7_27964696, S7_26602572, S7_25064701, S7_24676371, S7_24437217, S7_26607710, S7_24616764, S7_26172187, S7_23816490, [S7_23816504], S7_31316668, [S7_31316667], S7_26781102, S7_24746168, S7_23854494, S7_28206490, S7_27153605, S7_26731565, S7_31259191, [S7_31259192], S7_26403443, S7_26708171, S7_27222312, S7_24534142, S7_25170788, [S7_25170803], S7_23917495, S7_25862928, S7_24229859, S7_24834728, S7_24674484, S7_26077288, S7_26717863, S7_28540366, S7_31206204, S7_24412148, S7_25060560, S7_26287791, [S7_27972532], [S7_27972510], S7_26081545, S7_25109588, S7_25805997, [S7_27915324], S7_25170722, S7_26496445, S7_26496480, S7_28302885
S7_30306252
S7_29887798
S7_28883340
S7_28691304
S7_10382718, S7_10892260, S7_10937993, S7_11059677, S7_10892197, S7_10979775, S7_11166540, S7_10152438, S7_11019191, [S7_11019177], S7_28836340, [S7_28836343]

Chr-8

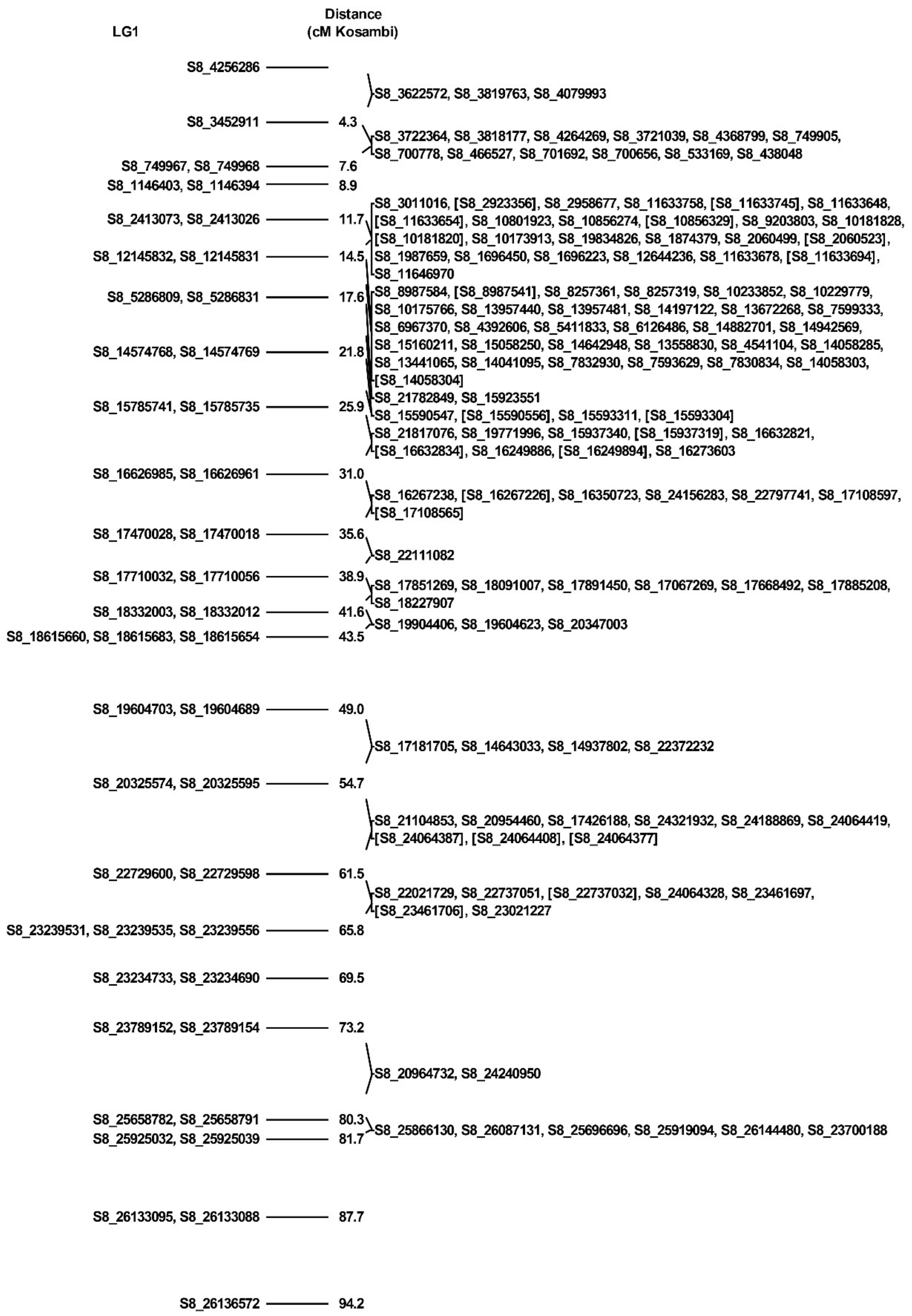
LG1
Distance
(cM Kosambi)
S8_4256286
S8_3622572, S8_3819763, S8_4079993
S8_3452911 4.3
S8_3722364, S8_3818177, S8_4264269, S8_3721039, S8_4368799, S8_749905,
S8_700778, S8_466527, S8_701692, S8_700656, S8_533169, S8_438048
S8_749967, S8_749968 7.6
S8_1146403, S8_1146394 8.9
S8_3011016, [S8_2923356], S8_2958677, S8_11633758, [S8_11633745], S8_11633648,
S8_2413073, S8_2413026 11.7
[S8_11633654], S8_10801923, S8_10856274, [S8_10856329], S8_9203803, S8_10181828,
[S8_10181820], S8_10173913, S8_19834826, S8_1874379, S8_2060499, [S8_2060523],
S8_12145832, S8_12145831 14.5
S8_1987659, S8_1696450, S8_1696223, S8_12644236, S8_11633678, [S8_11633694],
S8_11646970
S8_5286809, S8_5286831 17.6
S8_8987584, [S8_8987541], S8_8257361, S8_8257319, S8_10233852, S8_10229779,
S8_10175766, S8_13957440, S8_13957481, S8_14197122, S8_13672268, S8_7599333,
S8_6967370, S8_4392606, S8_5411833, S8_6126486, S8_14882701, S8_14942569,
S8_14574768, S8_14574769 21.8
S8_15160211, S8_15058250, S8_14642948, S8_13558830, S8_4541104, S8_14058285,
S8_13441065, S8_14041095, S8_7832930, S8_7593629, S8_7830834, S8_14058303,
[S8_14058304]
S8_21782849, S8_15923551
S8_15785741, S8_15785735 25.9
S8_15590547, [S8_15590556], S8_15593311, [S8_15593304]
S8_21817076, S8_19771996, S8_15937340, [S8_15937319], S8_16632821,
[S8_16632834], S8_16249886, [S8_16249894], S8_16273603
S8_16626985, S8_16626961 31.0
S8_16267238, [S8_16267226], S8_16350723, S8_24156283, S8_22797741, S8_17108597,
[S8_17108565]
S8_17470028, S8_17470018 35.6
S8_22111082
S8_17710032, S8_17710056 38.9
S8_17851269, S8_18091007, S8_17891450, S8_17067269, S8_17668492, S8_17885208,
S8_18227907
S8_18332003, S8_18332012 41.6
S8_19904406, S8_19604623, S8_20347003
S8_18615660, S8_18615683, S8_18615654 43.5
S8_19604703, S8_19604689 49.0
S8_17181705, S8_14643033, S8_14937802, S8_22372232
S8_20325574, S8_20325595 54.7
S8_21104853, S8_20954460, S8_17426188, S8_24321932, S8_24188869, S8_24064419,
[S8_24064387], [S8_24064408], [S8_24064377]
S8_22729600, S8_22729598 61.5
S8_22021729, S8_22737051, [S8_22737032], S8_24064328, S8_23461697,
[S8_23461706], S8_23021227
S8_23239531, S8_23239535, S8_23239556 65.8
S8_23234733, S8_23234690 69.5
S8_23789152, S8_23789154 73.2
S8_20964732, S8_24240950
S8_25658782, S8_25658791 80.3
S8_25866130, S8_26087131, S8_25696696, S8_25919094, S8_26144480, S8_23700188
S8_25925032, S8_25925039 81.7
S8_26133095, S8_26133088 87.7
S8_26136572 94.2

Chr-9

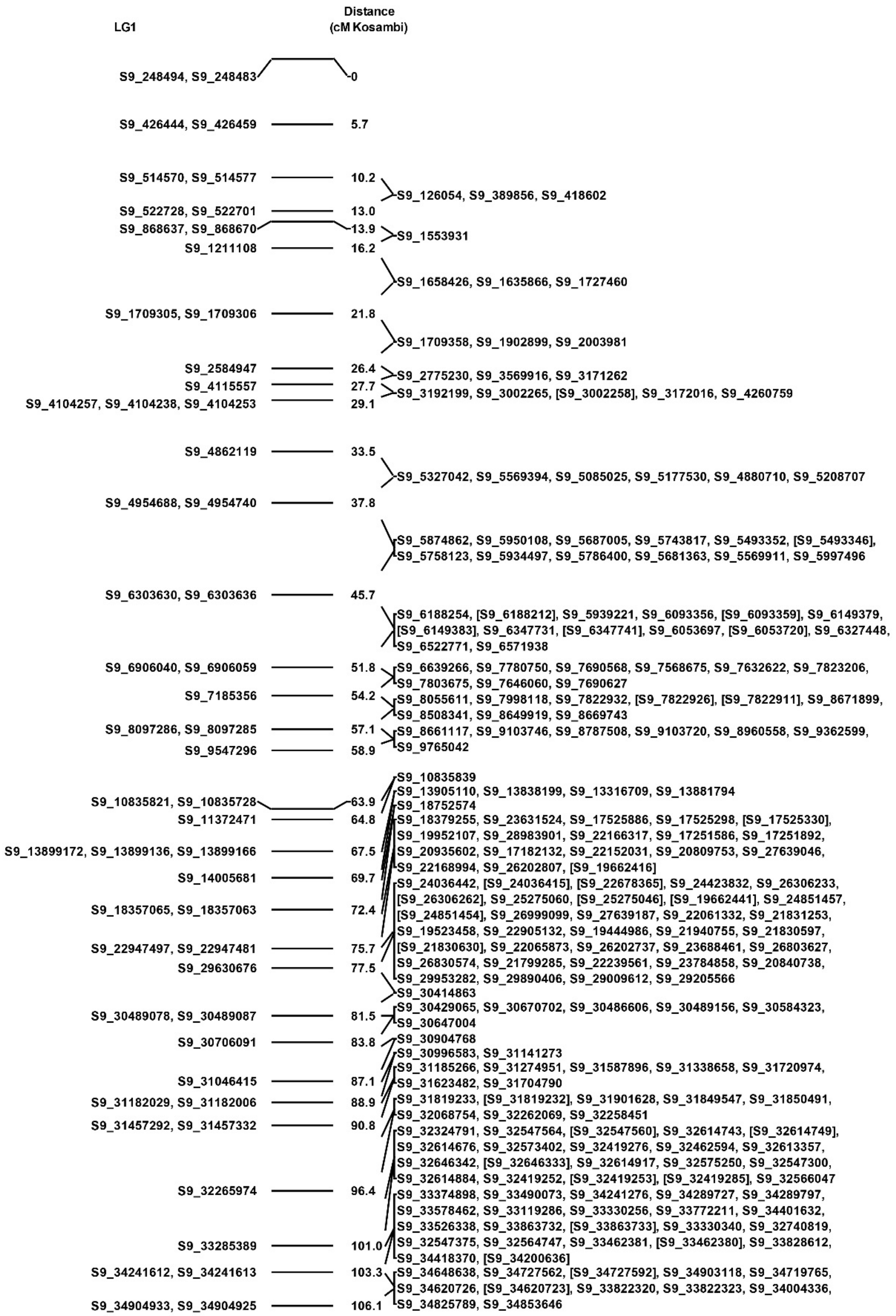

LG1
Distance
(cM Kosambi)
S9_248494, S9_248483
0
S9_426444, S9_426459
5.7
S9_514570, S9_514577
10.2
S9_126054, S9_389856, S9_418602
S9_522728, S9_522701
13.0
S9_868637, S9_868670
13.9
S9_1553931
S9_1211108
16.2
S9_1658426, S9_1635866, S9_1727460
S9_1709305, S9_1709306
21.8
S9_1709358, S9_1902899, S9_2003981
S9_2584947
26.4
S9_2775230, S9_3569916, S9_3171262
S9_4115557
27.7
S9_3192199, S9_3002265, [S9_3002258], S9_3172016, S9_4260759
S9_4104257, S9_4104238, S9_4104253
29.1
S9_4862119
33.5
S9_5327042, S9_5569394, S9_5085025, S9_5177530, S9_4880710, S9_5208707
S9_4954688, S9_4954740
37.8
S9_5874862, S9_5950108, S9_5687005, S9_5743817, S9_5493352, [S9_5493346],
S9_5758123, S9_5934497, S9_5786400, S9_5681363, S9_5569911, S9_5997496
S9_6303630, S9_6303636
45.7
S9_6188254, [S9_6188212], S9_5939221, S9_6093356, [S9_6093359], S9_6149379,
[S9_6149383], S9_6347731, [S9_6347741], S9_6053697, [S9_6053720], S9_6327448,
S9_6522771, S9_6571938
S9_6906040, S9_6906059
51.8
S9_6639266, S9_7780750, S9_7690568, S9_7568675, S9_7632622, S9_7823206,
S9_7803675, S9_7646060, S9_7690627
S9_7185356
54.2
S9_8055611, S9_7998118, S9_7822932, [S9_7822926], [S9_7822911], S9_8671899,
S9_8508341, S9_8649919, S9_8669743
S9_8097286, S9_8097285
57.1
S9_8661117, S9_9103746, S9_8787508, S9_9103720, S9_8960558, S9_9362599,
S9_9765042
S9_9547296
58.9
S9_10835839
S9_13905110, S9_13838199, S9_13316709, S9_13881794
S9_10835821, S9_10835728
63.9
S9_18752574
S9_11372471
64.8
S9_18379255, S9_23631524, S9_17525886, S9_17525298, [S9_17525330],
S9_19952107, S9_28983901, S9_22166317, S9_17251586, S9_17251892,
S9_13899172, S9_13899136, S9_13899166
67.5
S9_20935602, S9_17182132, S9_22152031, S9_20809753, S9_27639046,
S9_22168994, S9_26202807, [S9_19662416]
S9_14005681
69.7
S9_24036442, [S9_24036415], [S9_22678365], S9_24423832, S9_26306233,
[S9_26306262], S9_25275060, [S9_25275046], [S9_19662441], S9_24851457,
S9_18357065, S9_18357063
72.4
[S9_24851454], S9_26999099, S9_27639187, S9_22061332, S9_21831253,
S9_19523458, S9_22905132, S9_19444986, S9_21940755, S9_21830597,
S9_22947497, S9_22947481
75.7
[S9_21830630], S9_22065873, S9_26202737, S9_23688461, S9_26803627,
S9_29630676
77.5
S9_26830574, S9_21799285, S9_22239561, S9_23784858, S9_20840738,
S9_29953282, S9_29890406, S9_29009612, S9_29205566
S9_30414863
S9_30429065, S9_30670702, S9_30486606, S9_30489156, S9_30584323,
S9_30489078, S9_30489087
81.5
S9_30647004
S9_30706091
83.8
S9_30904768
S9_30996583, S9_31141273
S9_31185266, S9_31274951, S9_31587896, S9_31338658, S9_31720974,
S9_31046415
87.1
S9_31623482, S9_31704790
S9_31182029, S9_31182006
88.9
S9_31819233, [S9_31819232], S9_31901628, S9_31849547, S9_31850491,
S9_32068754, S9_32262069, S9_32258451
S9_31457292, S9_31457332
90.8
S9_32324791, S9_32547564, [S9_32547560], S9_32614743, [S9_32614749],
S9_32614676, S9_32573402, S9_32419276, S9_32462594, S9_32613357,
S9_32646342, [S9_32646333], S9_32614917, S9_32575250, S9_32547300,
S9_32614884, S9_32419252, [S9_32419253], [S9_32419285], S9_32566047
S9_32265974
96.4
S9_33374898, S9_33490073, S9_34241276, S9_34289727, S9_34289797,
S9_33578462, S9_33119286, S9_33330256, S9_33772211, S9_34401632,
S9_33526338, S9_33863732, [S9_33863733], S9_33330340, S9_32740819,
S9_33285389
101.0
S9_32547375, S9_32564747, S9_33462381, [S9_33462380], S9_33828612,
S9_34418370, [S9_34200636]
S9_34241612, S9_34241613
103.3
S9_34648638, S9_34727562, [S9_34727592], S9_34903118, S9_34719765,
S9_34620726, [S9_34620723], S9_33822320, S9_33822323, S9_34004336,
S9_34904933, S9_34904925
106.1
S9_34825789, S9_34853646

Chr-10

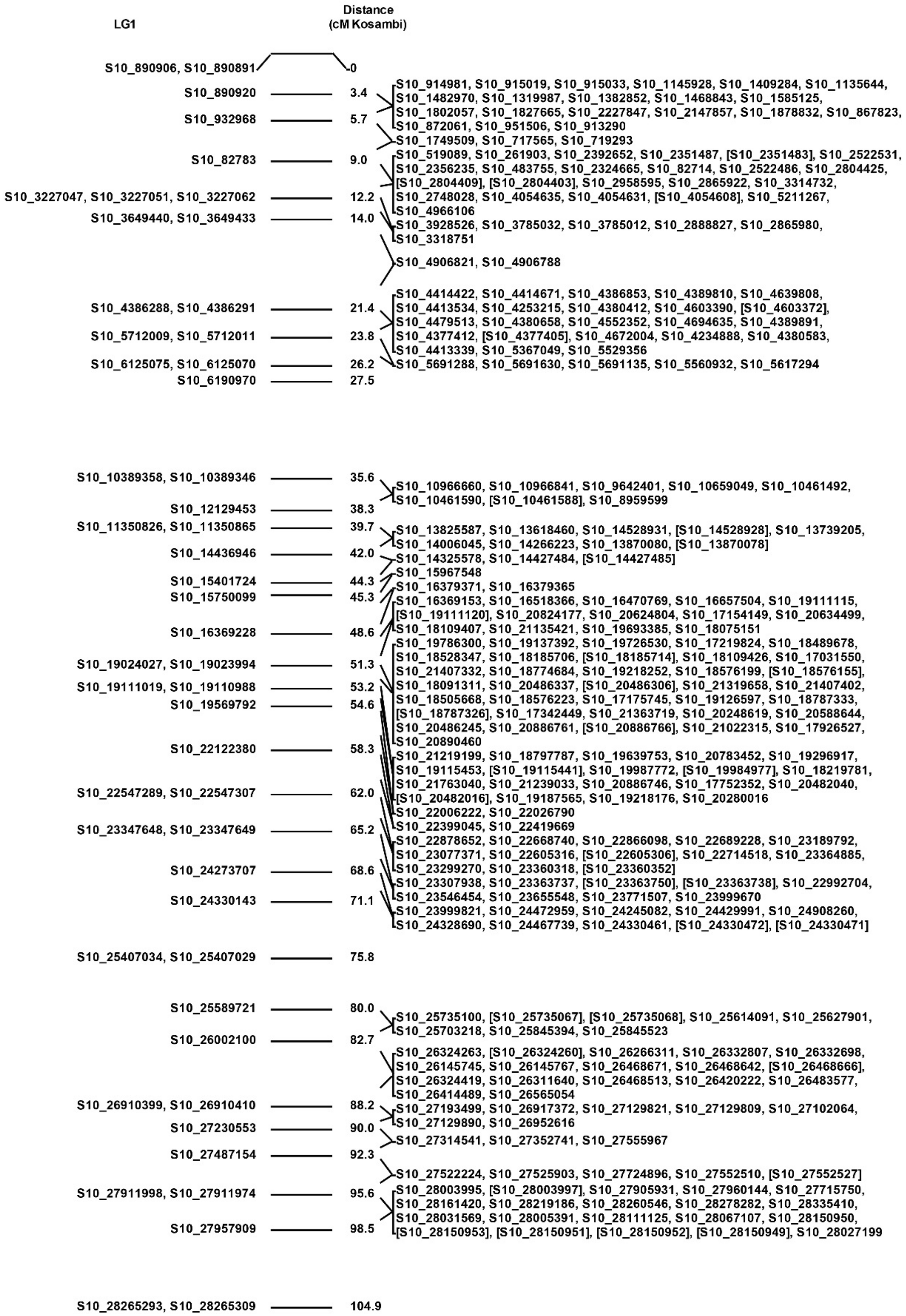
LG1
Distance
(cM Kosambi)
S10_890906, S10_890891 0
S10_890920 3.4
S10_932968 5.7
S10_82783 9.0
S10_3227047, S10_3227051, S10_3227062 12.2
S10_3649440, S10_3649433 14.0
S10_4386288, S10_4386291 21.4
S10_5712009, S10_5712011 23.8
S10_6125075, S10_6125070 26.2
S10_6190970 27.5
S10_10389358, S10_10389346 35.6
S10_12129453 38.3
S10_11350826, S10_11350865 39.7
S10_14436946 42.0
S10_15401724 44.3
S10_15750099 45.3
S10_16369228 48.6
S10_19024027, S10_19023994 51.3
S10_19111019, S10_19110988 53.2
S10_19569792 54.6
S10_22122380 58.3
S10_22547289, S10_22547307 62.0
S10_23347648, S10_23347649 65.2
S10_24273707 68.6
S10_24330143 71.1
S10_25407034, S10_25407029 75.8
S10_25589721 80.0
S10_26002100 82.7
S10_26910399, S10_26910410 88.2
S10_27230553 90.0
S10_27487154 92.3
S10_27911998, S10_27911974 95.6
S10_27957909 98.5
S10_28265293, S10_28265309 104.9
S10_914981, S10_915019, S10_915033, S10_1145928, S10_1409284, S10_1135644,
S10_1482970, S10_1319987, S10_1382852, S10_1468843, S10_1585125,
S10_1802057, S10_1827665, S10_2227847, S10_2147857, S10_1878832, S10_867823,
S10_872061, S10_951506, S10_913290
S10_1749509, S10_717565, S10_719293
S10_519089, S10_261903, S10_2392652, S10_2351487, [S10_2351483], S10_2522531,
S10_2356235, S10_483755, S10_2324665, S10_82714, S10_2522486, S10_2804425,
[S10_2804409], [S10_2804403], S10_2958595, S10_2865922, S10_3314732,
S10_2748028, S10_4054635, S10_4054631, [S10_4054608], S10_5211267,
S10_4966106
S10_3928526, S10_3785032, S10_3785012, S10_2888827, S10_2865980,
S10_3318751
S10_4906821, S10_4906788
S10_4414422, S10_4414671, S10_4386853, S10_4389810, S10_4639808,
S10_4413534, S10_4253215, S10_4380412, S10_4603390, [S10_4603372],
S10_4479513, S10_4380658, S10_4552352, S10_4694635, S10_4389891,
S10_4377412, [S10_4377405], S10_4672004, S10_4234888, S10_4380583,
S10_4413339, S10_5367049, S10_5529356
S10_5691288, S10_5691630, S10_5691135, S10_5560932, S10_5617294
S10_10966660, S10_10966841, S10_9642401, S10_10659049, S10_10461492,
S10_10461590, [S10_10461588], S10_8959599
S10_13825587, S10_13618460, S10_14528931, [S10_14528928], S10_13739205,
S10_14006045, S10_14266223, S10_13870080, [S10_13870078]
S10_14325578, S10_14427484, [S10_14427485]
S10_15967548
S10_16379371, S10_16379365
S10_16369153, S10_16518366, S10_16470769, S10_16657504, S10_19111115,
[S10_19111120], S10_20824177, S10_20624804, S10_17154149, S10_20634499,
S10_18109407, S10_21135421, S10_19693385, S10_18075151
S10_19786300, S10_19137392, S10_19726530, S10_17219824, S10_18489678,
S10_18528347, S10_18185706, [S10_18185714], S10_18109426, S10_17031550,
S10_21407332, S10_18774684, S10_19218252, S10_18576199, [S10_18576155],
S10_18091311, S10_20486337, [S10_20486306], S10_21319658, S10_21407402,
S10_18505668, S10_18576223, S10_17175745, S10_19126597, S10_18787333,
[S10_18787326], S10_17342449, S10_21363719, S10_20248619, S10_20588644,
S10_20486245, S10_20886761, [S10_20886766], S10_21022315, S10_17926527,
S10_20890460
S10_21219199, S10_18797787, S10_19639753, S10_20783452, S10_19296917,
S10_19115453, [S10_19115441], S10_19987772, [S10_19984977], S10_18219781,
S10_21763040, S10_21239033, S10_20886746, S10_17752352, S10_20482040,
[S10_20482016], S10_19187565, S10_19218176, S10_20280016
S10_22006222, S10_22026790
S10_22399045, S10_22419669
S10_22878652, S10_22668740, S10_22866098, S10_22689228, S10_23189792,
S10_23077371, S10_22605316, [S10_22605306], S10_22714518, S10_23364885,
S10_23299270, S10_23360318, [S10_23360352]
S10_23307938, S10_23363737, [S10_23363750], [S10_23363738], S10_22992704,
S10_23546454, S10_23655548, S10_23771507, S10_23999670
S10_23999821, S10_24472959, S10_24245082, S10_24429991, S10_24908260,
S10_24328690, S10_24467739, S10_24330461, [S10_24330472], [S10_24330471]
S10_25735100, [S10_25735067], [S10_25735068], S10_25614091, S10_25627901,
S10_25703218, S10_25845394, S10_25845523
S10_26324263, [S10_26324260], S10_26266311, S10_26332807, S10_26332698,
S10_26145745, S10_26145767, S10_26468671, S10_26468642, [S10_26468666],
S10_26324419, S10_26311640, S10_26468513, S10_26420222, S10_26483577,
S10_26414489, S10_26565054
S10_27193499, S10_26917372, S10_27129821, S10_27129809, S10_27102064,
S10_27129890, S10_26952616
S10_27314541, S10_27352741, S10_27555967
S10_27522224, S10_27525903, S10_27724896, S10_27552510, [S10_27552527]
S10_28003995, [S10_28003997], S10_27905931, S10_27960144, S10_27715750,
S10_28161420, S10_28219186, S10_28260546, S10_28278282, S10_28335410,
S10_28031569, S10_28005391, S10_28111125, S10_28067107, S10_28150950,
[S10_28150953], [S10_28150951], [S10_28150952], [S10_28150949], S10_28027199

Chr-11

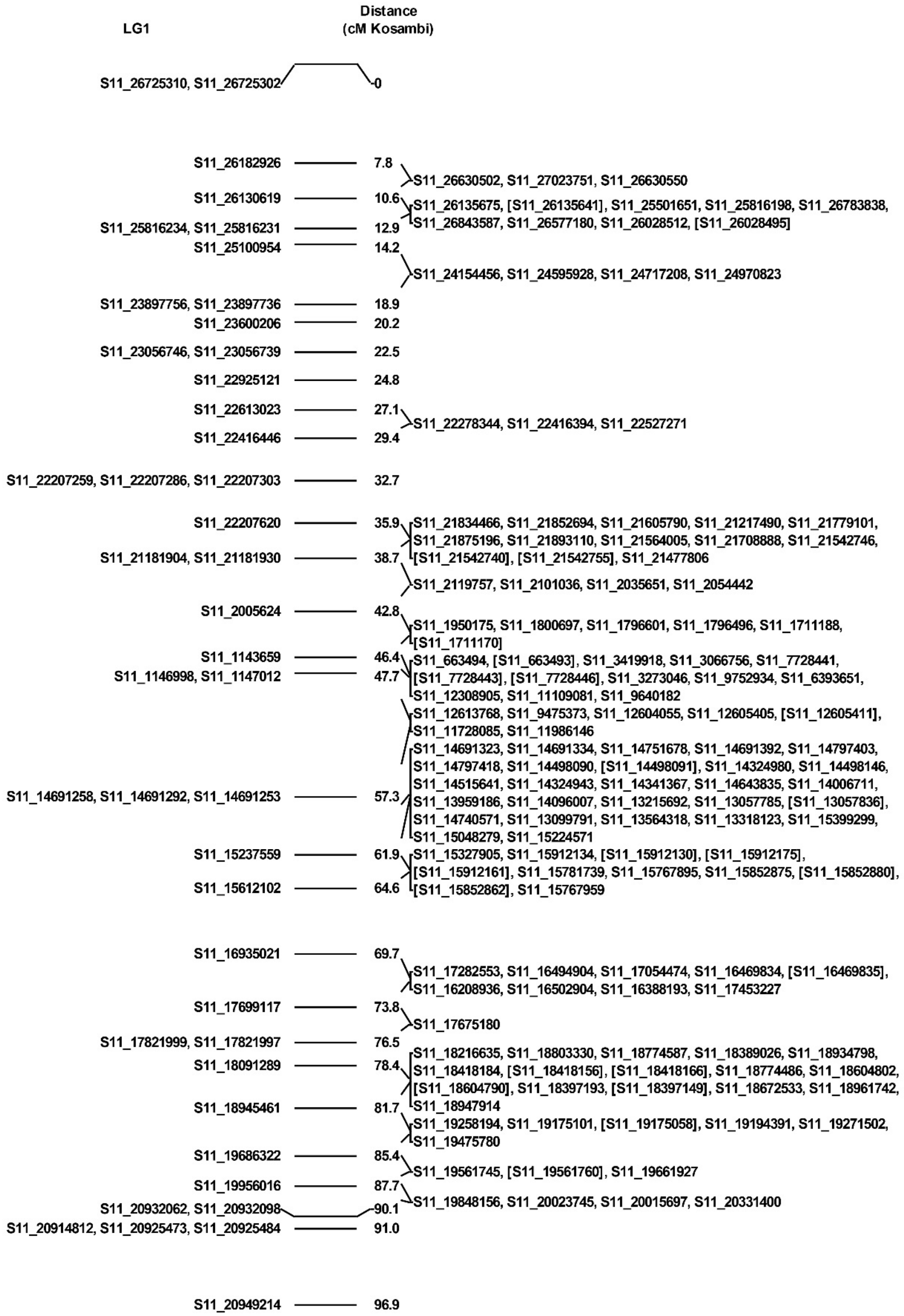

Figure S3 1 to 11: High-resolution genetic maps of various chromosomes consisting of add-on markers.

Tables S1-S13

Available for download as Excel files at http://www.g3journal.org/lookup/suppl/doi:10.1534/g3.114.012815/-/DC1

Table S1 Details of various accessions used in the study.

Table S2 Eigen values of first 2 vectors for various accessions used for principal component analysis.

Table S3 Add-on markers mapped to chromosome 1.

Table S4 Add-on markers mapped to chromosome 2.

Table S5 Add-on markers mapped to chromosome 3.

Table S6 Add-on markers mapped to chromosome 4.

Table S7 Add-on markers mapped to chromosome 5.

Table S8 Add-on markers mapped to chromosome 6.

Table S9 Add-on markers mapped to chromosome 7.

Table S10 Add-on markers mapped to chromosome 8.

Table S11 Add-on markers mapped to chromosome 9.

Table S12 Add-on markers mapped to chromosome 10.

Table S13 Add-on markers mapped to chromosome 11.
