# Supplementary material for: High-Resolution Genetic Map for Understanding the Effect of Genome-Wide Recombination Rate on Nucleotide Diversity in Watermelon
Source: G3 (Bethesda). 2014 Sep 15;4(11):2219–30. doi: 10.1534/g3.114.012815 (PMC4232547; doi:10.1534/g3.114.012815)
Supplement: Supporting Information [file supp_g3.114.012815_FigureS1.pdf]

**A.**

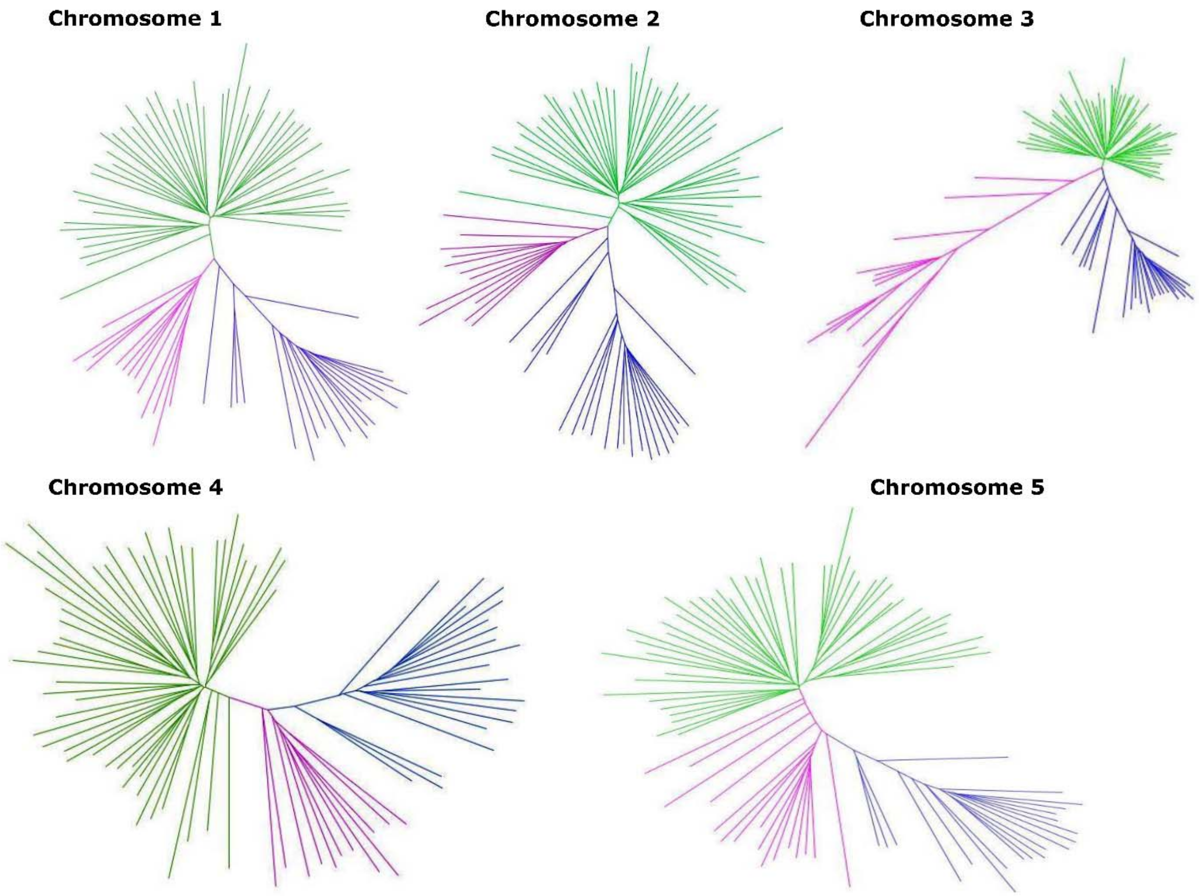
Chromosome 1
Chromosome 2
Chromosome 3
Chromosome 4
Chromosome 5

**B.**

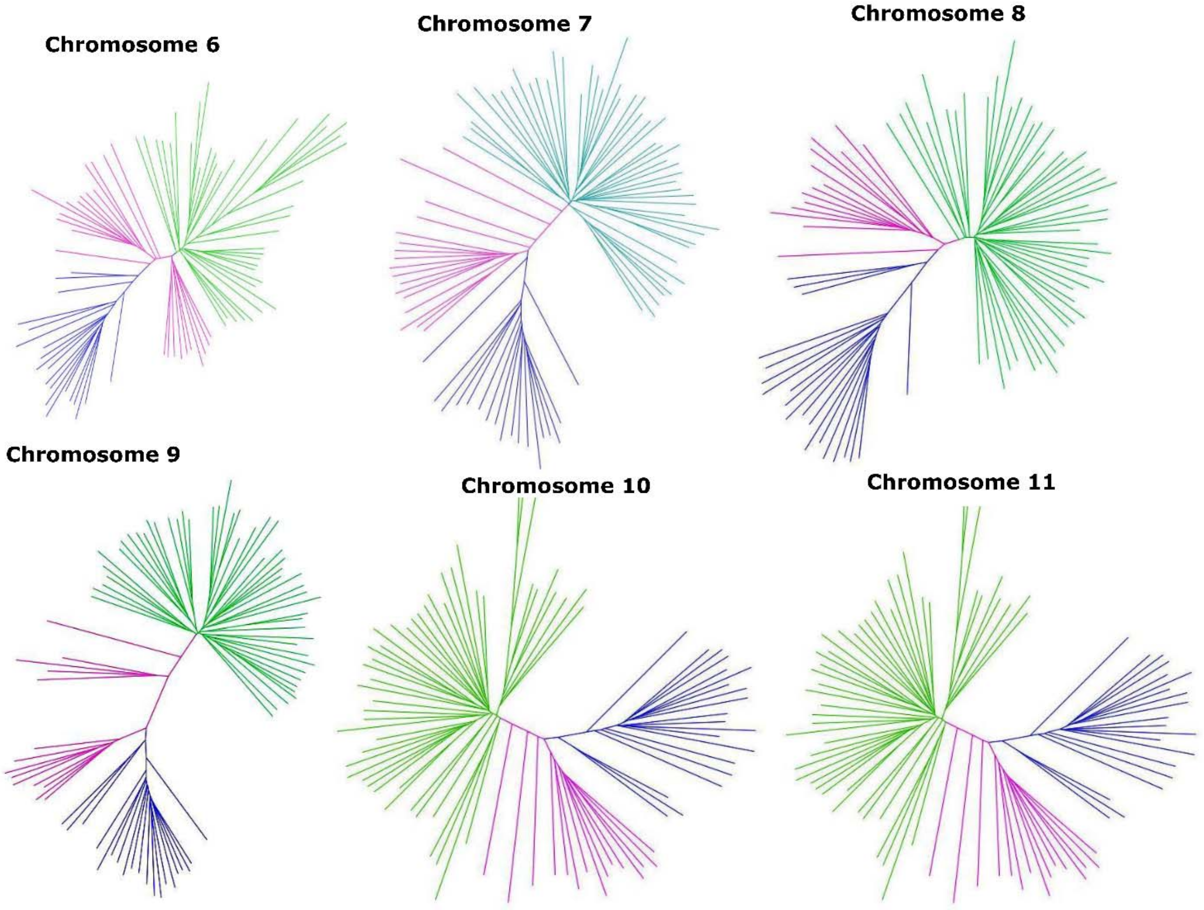


**Figure S1** A and B: Chromosome-wise neighbor-joining trees for sweet, semi-wild and wild watermelon (Green: sweet, pink: semi-wild, blue: wild)
