# Supplementary material for: High-Resolution Genetic Map for Understanding the Effect of Genome-Wide Recombination Rate on Nucleotide Diversity in Watermelon
Source: G3 (Bethesda). 2014 Sep 15;4(11):2219–30. doi: 10.1534/g3.114.012815 (PMC4232547; doi:10.1534/g3.114.012815)
Supplement: Supporting Information [file supp_g3.114.012815_FigureS2.pdf]

Burnin iterations = 100000
MCMC iterations = 500000
Replications = 4

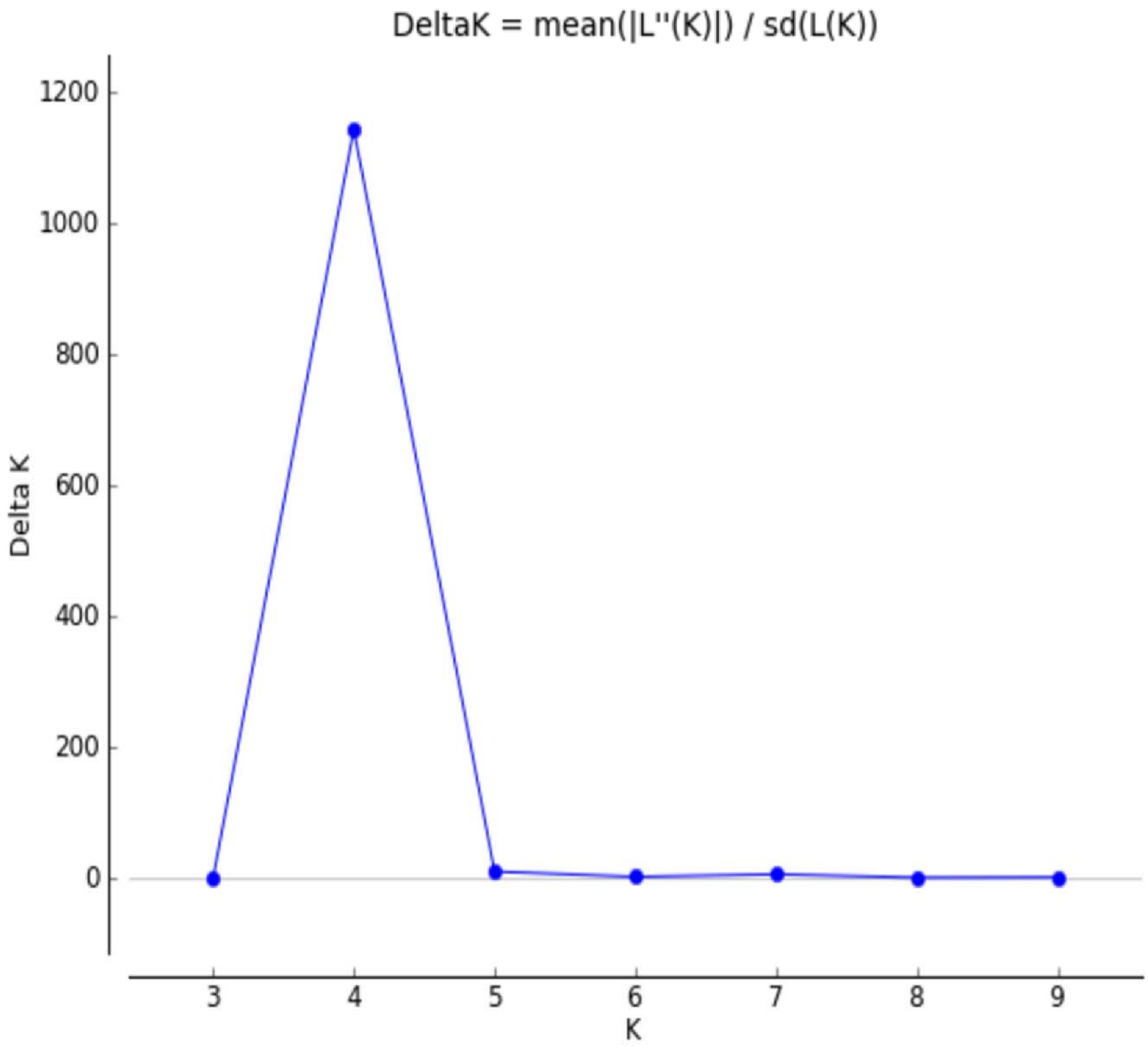

Figure S2 ΔK distribution for various clusters estimated by use of Structure Harvester. Highest ΔK value indicate the optimum cluster (K) for the current watermelon population.
